# A neutrophil mimicking metal-porphyrin-based nanodevice loaded with porcine pancreatic elastase for cancer therapy

**DOI:** 10.1038/s41467-023-37580-z

**Published:** 2023-04-08

**Authors:** Tingting Cui, Yu Zhang, Geng Qin, Yue Wei, Jie Yang, Ying Huang, Jinsong Ren, Xiaogang Qu

**Affiliations:** 1 State Key Laboratory of Rare Earth Resource Utilization and Laboratory of Chemical Biology, Changchun Institute of Applied Chemistry, Chinese Academy of Sciences, Changchun, Jilin 130022 P. R. China; 2 School of Applied Chemistry and Engineering, University of Science and Technology of China, Hefei, Anhui 230026 P. R. China

**Keywords:** Immunochemistry, Cancer immunotherapy, Biomedical materials

## Abstract

Precise discrimination and eradication of cancer cells by immune cells independent of antigen recognition is promising for solid tumor therapeutics, yet remains a tremendous challenge. Inspired by neutrophils, here we design and construct a tumor discrimination nanodevice based on the differential histone H1 isoform expression. In this nanodevice, neutrophil membrane camouflage and glutathione (GSH)-unlocking effect on Fe-porphyrin metal−organic framework structure ensures selectivity to cancer cells. The released porcine pancreatic elastase (PPE) simulates neutrophils’ action to induce histone H1 release-dependent selective cancer cell killing. Meanwhile, nuclear localization signal (NLS) peptide-tagged porphyrin (porphyrin-NLS) acts as in-situ singlet oxygen (^1^O_2_) generator to amplify histone H1 nucleo-cytoplasmic translocation by inducing DNA double-strand breaks (DSBs) under laser irradiation, further promoting elimination of cancer cells. The overexpressed histone H1 isoform in cancer cells improves selectivity of our nanodevice to cancer cells. In vivo studies demonstrate that our design can not only inhibit primary tumor growth, but also induce adaptive T-cell response-mediated abscopal effect to against distal tumors.

## Abstract (web summary)

Porcine neutrophil elastase (PPE) has been demonstrated to have a histone H1 isoform dependent anti-tumour effect. Here, the authors develop a biomimetic nanoplatform combining PPE and elevated histone H1 release induced by porphyrin nuclear localisation signal peptide and demonstrate its efficacy in preclinical cancer models.

## Introduction

Precision tumor discrimination and eradication is a prerequisite for successful cancer therapies^1,2^. Immune cells, as the key players in the host defense system, have evolved the remarkable capability to traffic through the body, recognize and kill tumor cells^3–5^. By harnessing these intrinsic properties of immune cells, researchers have developed cell-based immunotherapy as a highly promising therapeutic modality to meet requirements for tumor recognition^6–9^. For example, T cells, NK cells, and macrophages have been genetically engineered with chimeric antigen receptors (CARs) to enhance their cytotoxic activity toward cancer cells^10–14^. Using cell surface engineering techniques to display natural and artificial receptors on immnue cells can also boost tumor cell-targeting therapies^15–18^. Despite great potential, these engineered immune cell strategies have several limitations^19^. The cells need to be engineered with different antigen receptors or ligands to directly against various tumors with antigen heterogeneity^20^. Such a pattern is time and resource intensive, and not suitable as broad-spectrum anticancer therapy^8^. More importantly, few antigens are absolutely specific to tumors^21^. Most targeted antigens are also present in normal tissues where toxic “off-tumor” cross-reaction with engineered immnue cells can occur, causing life-threatening adverse side effects^22^. Therefore, developing other tactics by immune cells independent of antigen recognition is highly desirable for precise cancer therapy.

Neutrophils, the most prevalent innate immunity effector cells, are of particular interest due to their capacity for inflammation targeting and pathogen elimination^23–25^. Very recent works have demonstrated that neutrophil-released neutrophil elastase (ELANE) or its homolog (porcine pancreatic elastase (PPE)) can kill many kinds of cancer cell types (35 different cancer cell lines) while preserving non-cancer cells and trigger an abscopal effect mediated by cytotoxic T lymphocytes to attenuate primary and distal tumor growth^26,27^. The crucial factor driving broad specificity for cancer cells is the selective expression of histone H1 isoforms in numerous tumor types. Mechanistically, ELANE or PPE enters the cell and proteolytically liberates the CD95 death domain (DD). Then CD95 DD induces DNA damage, provoking histone H1 isoform nucleo-cytoplasmic translocation to bind CD95 DD and subsequently co-localize with mitochondria^28^. This finally initiates apoptotic pathway in mitochondria, killing cancer cells and liberating cancer-specific antigens that elicit adaptive immune response in vivo. The ELANE or PPE’s cancer-selective property is dependent on the elevated histone H1 isoforms but not the rare homogeneously expressed and tumor-restricted antigens, a property that raises the possibility of developing them as powerful tumor-agnostic and mutation-agnostic targeted agents^29^. Onchilles Pharma has been exploited ELANE and PPE therapies for clinical use^30^. Considering the advantages of neutrophils and engineered immune cell strategies, adoptive transfer of engineered neutrophils to release ELANE appears to be an effective anticancer strategy. However, neutrophil-based therapies face several challenges, such as uncertain phenotypes and functional variations in neutrophils, significantly low delivery efficacy due to the majority of systemically administered cells being trapped in the lungs, and the complexity of manipulation and storage of live neutrophils^31–33^.

The cell-free biomimetic strategy bridges the gap between cell and nanomaterials, offering potential to circumvent the problems encountered by cell-based therapies^32,34–40^. By integrating the tumor-homing properties of immune cells and user-defined functional responses of nanomaterials, this strategy enables a synthetic system to effectively transport therapeutic payloads to tumor sites and achieve spatiotemporal control and on-demand intracellular release^41–43^. However, despite the approach promising, unsatisfactory cancer cell-killing effect and inadequate immune activation of ELANE/PPE alone limit its potential application in tumor therapy. Amplifying tumor cell killing activity of ELANE/PPE while retaining precise targeting capacity is needed to achieve maximal therapeutic effect.

Considering that the nucleo-cytoplasmic translocation of histone H1 plays a crucial role in precision tumor discrimination and killing effect of ELANE/PPE, we hypothesized that accelerating histone H1 translocation would enable maximal therapeutic effect. As we all know, histone H1 binds to the linker DNA between nucleosomes and seals off two turns of DNA around a histone octamer, which can protect DNA from DNA-damaging agents^44,45^. Note that once DNA double-strand breaks (DSBs) occur, a significant amount of Histone H1 is released from the chromatin, which in turn increases the sensitivity of DNA to DNA DSB inducers, resulting in more DSBs^45–47^. Previous studies showed that reactive oxygen species (ROS), especially singlet oxygen (^1^O_2_), can cause DSBs^39^. Thus, we envisioned that introducing a ^1^O_2_ generator that produces ^1^O_2_ only in cancer cells into the therapeutic system could enhance selective cancer cell killing.

**Fig. 1:**
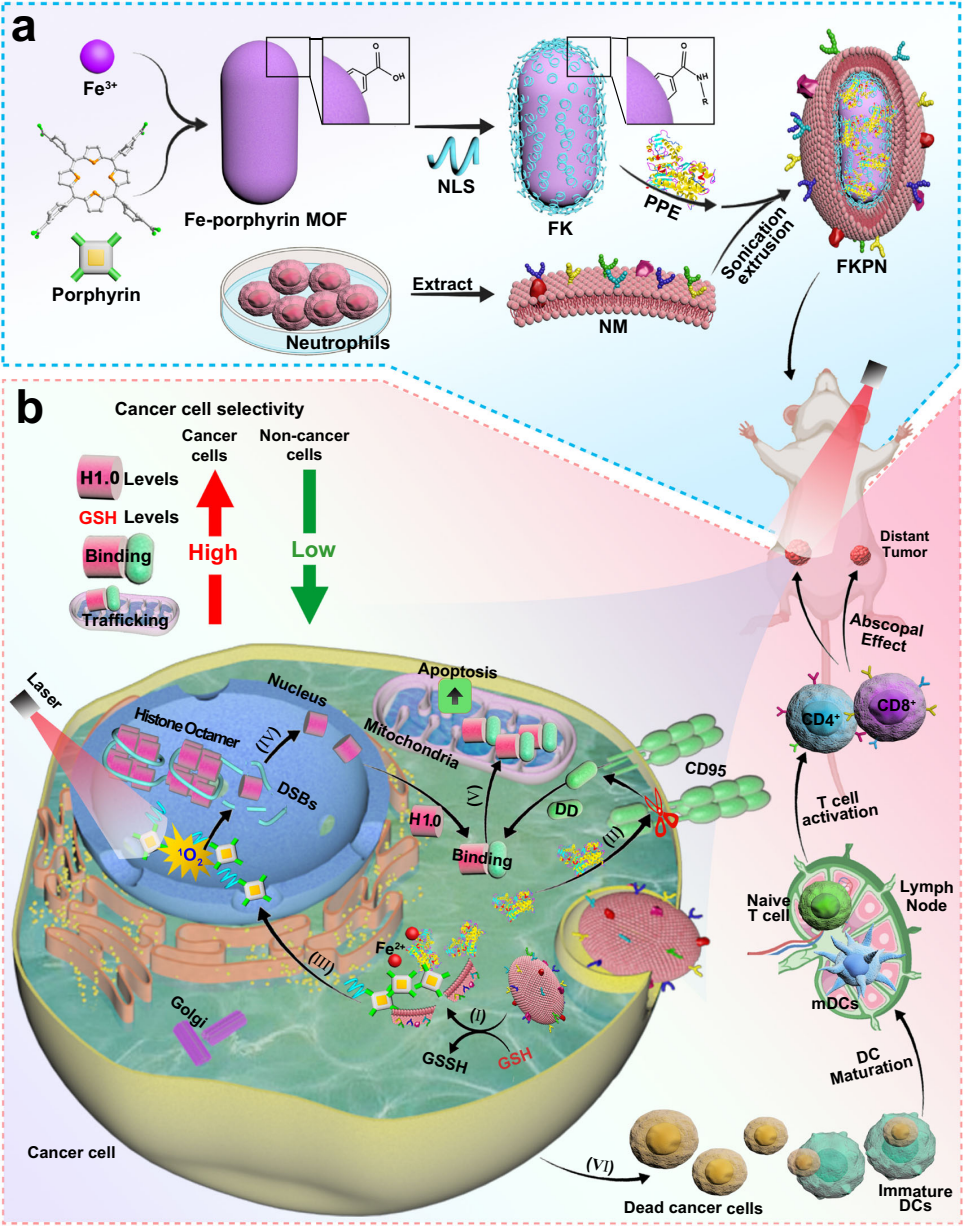
Tumor-specific amplification of histone H1 translocation by FKPN mediates precision tumor discrimination and cancer immunotherapy. **a** Schematic illustration of preparation of biomimetic nanodevice FKPN. **b** Biological mechanism of FKPN for selective cancer cell killing and immune system activation through PPE and tumor-specific nuclear ^1^O_2_ generator: (I) GSH-triggered FKPN unlocking on porphyrin-NLS and PPE release; (II) PPE-mediated proteolytic liberation of CD95 DD; (III) nuclear transport and photodynamic effect of porphyrin-NLS; (IV) Amplified histone H1 isoform release through nuclear ^1^O_2_ generation under laser irradiation; (V) CD95 DD-H1.0 interactions and subsequent mitochondrial trafficking triggers apoptosis pathways; (VI) The enhanced cancer cell apoptosis induce DC maturation and increase CD4^+^ and CD8^+^ T-cell infiltration into tumor tissues for primary and distant tumor eradication. NLS: nuclear localization signal; FK: Fe-porphyrin MOF-NLS; PPE: porcine pancreatic elastase; NM: neutrophil membrane; H1.0: histone H1 isoforms; DD: death domain; GSH: glutathione; GSSH: oxidized glutathione; DSBs: DNA double-strand breaks; ^1^O_2_: singlet oxygen; DCs: dendritic cells; mDCs: mature dendritic cells.

Here, we report a biomimetic nanodevice (FKPN) by integrating innate immune factor PPE, nuclear ^1^O_2_ generator porphyrin-NLS, metal node Fe^3+^, and neutrophil membrane (NM) inspired by an alternative cancer-targeting mechanism (Fig. 1a). The nanoscale metal-organic framework (MOF) structure and NM coating enable FKPN to target tumor sites (Fig. 1b)^48,49^. The synthesized MOF is then unlocked in cancer cells in response to high intracellular glutathione (GSH) to release porphyrin-NLS and PPE, which proteolytically liberates the CD95 DD in cytosol, causing moderate histone H1 translocation^50,51^. Meanwhile, porphyrin-NLS targets the nucleus and in situ generates ^1^O_2_ under laser irradiation to induce DSBs and promote histone H1 isoform release. Then, the translocated histone H1 binds to CD95 DD binding, this leads to eradication of cancer cells by triggering apoptosis pathways in mitochondrion. Tumor-specific amplification of histone H1 translocation mediates precision tumor discrimination and efficient killing by FKPN, limiting host toxicity. Besides, FKPN can induce an abscopal effect against distal tumors at the same time as eliminating primary tumors by activating adaptive immune response.

## Results

### FKPN preparation and characterization

**Fig. 2:**
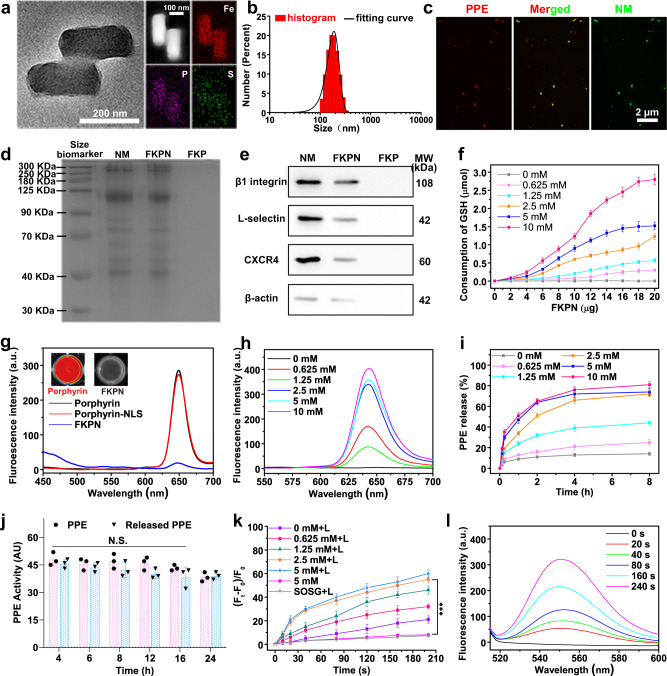
Characterization of FKPN. **a** TEM image, HAADF-STEM, and elemental mapping analysis of FKPN. **b** Size distribution of FKPN. **c** Fluorescence images of FKPN nanodevice. Green: Rhodamine 110 (Rh110)-labeled membrane; red: rhodamine B (RhB)-labeled PPE. **d** Protein profiles of membrane fragments, FKPN, and FKP characterized by SDS-PAGE. Gel is representative of *n* = 2 independent experiments. **e** Western blot results of representative adhesion proteins. Blot is representative of *n* = 2 independent experiments. **f** Quantification of GSH consumption using DTNB as an indicator. Data were presented as mean ± SD (*n* = 3 independent experiments). **g** Fluorescence spectrum of porphyrin, porphyrin-NLS, and FKPN. Inset: fluorescent images of porphyrin or FKPN in PBS; red: porphyrin fluorescent. **h** FKPN fluorescence changes with different GSH concentrations. **i** GSH-responsive release curve of PPE from FKPN treated by different concentrations of GSH. Data were presented as mean ± SD (*n* = 3 independent experiments). **j** Detection of PPE catalytic activity with *N*-methoxysuccinyl-Ala-Ala-Pro-Val p-nitroanilide (pNA). **k** Quantification of ROS generation using SOSG probe under laser irradiation (0.3 W/cm^2^, 660 nm). Data were presented as mean ± SD (*n* = 3 independent experiments). *P* values: 2.26352E-5. *P* values were assessed using Student’s *t* test (two-tailed) (***p < 0.001). **l** Fluorescence spectra of the SOSG and FKPN in GSH solution (10 mM) with different irradiation times. **a**–**c** Representative of *n* = 3 independent experiments. Source data are provided as a Source Data file.

First, the Fe-porphyrin MOF was fabricated according to previous work with some modifications, and characterized by scanning electron microscopy (SEM), transmission electron microscope (TEM), UV-vis absorption spectra as well as Fourier transform infrared (FTIR) spectra (Supplementary Figs. 1 and 2). Then NLS peptides (KRRRRG) were covalently grafted to the free carboxylic groups from porphyrin ligands in Fe-porphyrin MOF (FK) via the classical two-step EDC/NHS method (Supplementary Fig. 3,4)^52^. Then the negatively charged PPE was adsorbed on the positively charged FK (FKP) through multivalent electrostatic interactions, as confirmed by the UV-vis absorption analysis (Supplementary Fig. 5). At the same time, decreased zeta potential, nitrogen adsorption volume, and pore volume, and increased hydrodynamic diameter of particles also verified the successful loading of protein on the surface and in the pores of FK (Supplementary Figs. 6–9). FK and FKP particle concentrations were determined by flow cytometer analysis (Supplementary Fig. 10). Using the bicinchoninic acid (BCA) assay, the peptide or PPE concentrations loaded on the FK and FKP particles were determined (Supplementary Fig. 11). Based on these data, the peptide number and PPE number on each FKP particle were evaluated at ca. 1328 and 422, respectively. Based on the thermogravimetric analysis (TGA) and BCA protein assay kit results, the PPE loading efficiency was about 10.27% (Supplementary Fig. 12). Next, NM fragments derived from purified and activated neutrophils were coated onto FKP by ultrasonic bath and repeated extrusion to obtained biomimetic nanodevice (FKPN) (Supplementary Fig. 13 and Fig. 2a). The FKPN exhibited a uniform size (approximately 240 nm in length and 95 nm in width) with monodispersity. High-angle annular dark-field scanning transmission electron microscopy (HAADF-STEM) and elemental mapping analysis showed the distribution of Fe, P, and S elements (Fig. 2a). The hydrodynamic diameter of FKPN was increased after membrane coating (Fig. 2b). Meanwhile, the zeta potential of FKPN was decreased from 11.7 mV to −16.2 mV, which was comparable to neutrophil membrane-derived vesicles (Supplementary Fig. 6). The colocalization in confocal laser scanning microscopy (CLSM) images and Pearson correlation analysis indicated over 90% of FKs were simultaneously loaded with PPE and camouflaged with membrane coating (Fig. 2c and Supplementary Fig. 14).

The hydrodynamic size distribution and morphology of FKPN were nearly constant in pH 7.4 PBS over 7 days, indicating that NM was sufficiently stable (Supplementary Fig. 15). Subsequently, the preservation of protein profiles in FKPN was verified by SDS-PAGE electrophoresis (Fig. 2d). Western blot assays showed the presence of representative adhesion proteins, including L-selectin, CXCR4 and β1 integrin on FKPN, further confirming the translocation of NM onto the MOF cores (Fig. 2e). Next, GSH-triggered unlocking of FKPN was validated. X-ray photoelectron spectroscopy (XPS) analysis showed that Fe 2*p*_3/2_ band of FKPN changed from the characteristic peak of Fe^3+^ to Fe^2+^ state after GSH treatment (Supplementary Fig. 16). In parallel, the morphology of FKPN was seriously destroyed with time when incubated with GSH (Supplementary Fig. 17). After 4 h of incubation with GSH, FKPN was almost completely disintegrated. Then 5,5′-dithiobis-2-(nitrobenzoic acid) (DTNB) was used to quantitatively analyze the GSH consumption (Fig. 2f). As the concentration of GSH and FKPN increased, the consumption of GSH increased rapidly. The fluorescence of FKPN was significantly quenched compared to that of free porphyrin and porphyrin-NLS (Fig. 2g). However, the fluorescence intensity of porphyrin exhibited a significant increase with increasing GSH concentrations (Fig. 2h). Consistent with porphyrin release, Fe^2+^ from particles showed a comparable release trend (Supplementary Fig. 18). These results indicated that the FKPN still remained rapid and sensitive GSH-responsiveness. We then evaluated the kinetics of PPE release accompanying FKPN disintegration after exposure to GSH. As expected, GSH treatment induced substantial release of PPE within 4 h and complete release in 6 h (Fig. 2i). More importantly, the released PPE retained nearly the same activity as that for the free enzyme over long periods of time (Fig. 2j). Finally, we investigated the GSH-regulated ^1^O_2_ generation using singlet oxygen sensor green (SOSG) as an indicator. FKPN alone exhibit negligible ^1^O_2_ signals under laser irradiation (0.3 W/cm^2^ at 660 nm, 3 min), indicating its high stability in the absence of GSH (Fig. 2k). In contrast, after adding GSH (2.5 mM, without excess amount) into FKPN solution for 3 h, the ^1^O_2_ signals was increased rapidly under the same conditions. Notably, upon laser irradiation, the ^1^O_2_ signal of FKPN with excess GSH (10 mM) was still significantly increased, indicating the ^1^O_2_ generation ability of FKPN even in a reduction environment (Fig. 2i).

### Cancer cell-specific histone H1 translocation

**Fig. 3:**
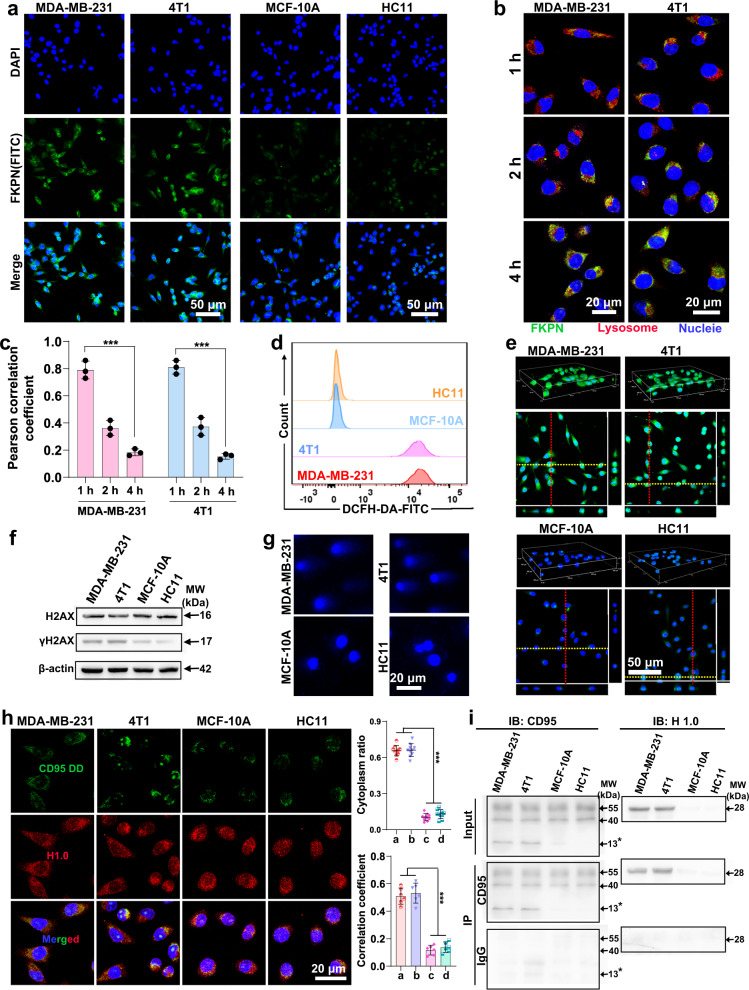
FKPN uptake by cancer cells mediates cancer cell-specific histone H1 translocation under laser irradiation. **a** Fluorescence images of cancer and non-cancer cells incubated with FKPN for 4 h. **b** CLSM images of MDA-MB-231 and 4T1 cells treated with FKPN for 1, 2, and 4 h. Blue: Hoechst 33342-labeled nuclei; red: LysoTracker red-labeled lysosomes; green: FITC-labeled FKPN. **c** The Pearson correlation coefficient of colocation of lysosomes and FKPN in **b**. Data were presented as mean ± SD (*n* = 3 independent experiments). *P* values: 0.000116295 for MDA-MB-231 cells treated with FKPN for 1 h vs for 4 h; 3.03889E-5 for 4T1 cells treated with FKPN for 1 h vs for 4 h. **d** Flow cytometry results of intracellular ^1^O_2_ levels in cancer and non-cancer cells after treatment with FKPN using DCFH-DA as a ^1^O_2_ fluorescent probe. **e** 3D-CLSM images of cancer and non-cancer cells after co-incubation with FKPN for 4 h and treated with laser irradiation; the corresponding CLSM photomicrograph with YZ (cells on the red line) and XZ (cells on the yellow line) planes of treated cells. DCFH-DA as a ^1^O_2_ detector. **f** Western blot results of γH2AX and H2AX protein in cancer and non-cancer cells after treatment with FKPN plus laser irradiation. Blot is representative of *n* = 2 independent experiments. **g** DNA damages of cancer and non-cancer cells in different groups detected by comet assays. **h** Immunofluorescence images of cancer and non-cancer cells stained for CD95 DD, H1.0, and nuclei after 4 h incubation with FKPN followed by laser irradiation and another 4 h incubation; corresponding calculations of cytoplasmic H1.0 ratio in treated cells; calculations of the correlation coefficient between CD95 DD and H1.0. **a** MDA-MB-231; **b** 4T1; **c** MCF-10A; **d** HC11. Data were presented as mean ± SD (*n* = 10 or 6 independent experiments). *P* values: 3.03418E-18 for cytoplasmic H1.0 ratio in MDA-MB-231 and 4T1 cells vs MCF-10A and HC11 cells; 1.22104E-12 for correlation coefficient of MDA-MB-231 and 4T1 cells vs MCF-10A and HC11 cells. **i** Co-immunoprecipitation analysis of CD95 DD with H1.0 in treated cancer and non-cancer cells. Blot is representative of *n* = 2 independent experiments. *P* values were assessed using Student’s *t* test (two-tailed) (***p < 0.001). **a**, **b** Representative of *n* = 3 independent experiments. Source data are provided as a Source Data file.

To explored whether the nanodevice can selectively amplify histone H1 translocation signals in cancer cell, we used MDA-MB-231 human breast cancer cell line, 4T1 murine breast cancer cell line, MCF-10A human normal breast epithelial cell line and HC11 murine normal breast epithelial cell line for the following examines. For better understanding how our designed FKPN to work, the control particles, FKN without PPE loading and FPN without NLS grafting, were synthesized, and their GSH-unlocking effects on payload release were comparable to that of FKPN group. Detailed experiment procedures and characterizations were provided in the supporting information (Supplementary Figs. 19–21). And the membrane coating did not obviously affect the release profile of PPE. Firstly, we encapsulated fluorescein isothiocyanate (FITC, green) into MOF structure to image the particles and different cell lines were incubated with various FITC-labeled particles to determine the cellular uptake. Fluorescence images exhibited that cancer cells treated with FKPN displayed a significantly enhanced green fluorescence in comparison to MCF-10A and HC11 cells (Fig. 3a and Supplementary Fig. 22). And the FKPN exhibited an approximately 3.0 times increased cellular uptake than FKP by MDA-MB-231 cells. These data indicated NM-mediated selective and enhanced cellular uptake of FKPN by MDA-MB-231 and 4T1 cells. Next, we studied the internalization mechanism of FKPN to explore the behaviors of FKPN in cancer cells. Previous studies revealed that glycoproteins such as CD44 were aberrantly expressed in cancer cells and act as ligands for selectins to facilitate tumor metastasis^43^. It has also been reported that the breast cancer cell lines can upregulate the expression of intracellular cell adhesion molecule-1 (ICAM-1) to mediate the attachment of neutrophils during the neutrophil recruitment to inflammation sites^33^. Flow cytometry results showed that glycoproteins CD44 and ICAM-1 were indeed upregulated on both MDA-MB-231 and 4T1 cells (Supplementary Fig. 23). Then we performed antibody blocking assay to evaluate the effect of membrane proteins on FKPN (Supplementary Figs. 23 and 24). CLSM images showed that the fluorescence intensity of FKPN in MDA-MB-231 was reduced by about 51.6% and 53.9% following preincubation with anti-CD44 and anti-ICAM-1 antibodies, respectively. Similarly, in 4T1 cells preincubated with anti-CD44 and anti-ICAM-1 antibodies, the fluorescence intensity was reduced by about 51.2% and 49.9%, respectively. These results indicated that the specific binding of aberrant overexpressed proteins on cancer cells with adhesion proteins on FKPN mediated targeting and cellular uptake of FKPN. Next, we preincubated 4T1 and MDA-MB-231 cells with low temperature (4 °C) and multiple endocytosis inhibitors, followed by adding FKPN nanoparticles (NPs) (Supplementary Fig. 25). Notably, the cellular uptake of FKPN was significantly inhibited at 4 °C, suggesting the energy-dependent internalization pathway of FKPN. The uptake efficiency of FKPN in MDA-MB-231 and 4T1 cells was reduced by about 79.5% and 80.8% respectively in the presence of chlorpromazine (CPZ, blocking clathrin-dependent endocytosis), while it was slightly affected after preincubation with other inhibitors, indicating that FKPN entered the cells primarily through clathrin-dependent endocytosis, commonly referred to as receptor-mediated endocytosis. Furthermore, CLSM images showed that excellent fluorescence merging of FKPN-incorporated FITC and lysosome-stained LysoTracker red after 1 h of incubation colocalization (Fig. 3b and Supplementary Fig. 26). After 2 h of incubation, FKPN began to diffuse to the cytoplasm as revealed by decreased fluorescence colocalization. At 4 h, a significant amount of FKPN localized outside the lysosomes, and the corresponding Pearson correlation coefficient decreased from 0.79 and 0.81 to 0.18 and 0.15, indicating effective escape of FKPN from lysosomes (Fig. 3c). Next, three-dimensional confocal laser scanning microscopy (3D-CLSM) images showed that cancer cells incubated with FKPN exhibited stronger intracellular fluorescence of porphyrin than MCF-10A and HC11 cells, indicating more cancer cell-selective disintegration of particles (Supplementary Fig. 27a). Importantly, FKPN and FKN treated MDA-MB-231 cells showed significantly higher nuclear localization than that of FPN treatment group, suggesting enhanced nucleus-targeting delivery of porphyrin-NLS (Supplementary Figs. 27b, c). Moreover, fluorescence images showed that FKPN and FPN significantly improved the internalization of fluorescein isothiocyanate (FITC, green)-labeled PPE compared with free PPE group in MDA-MB-231 cells, and the green fluorescence signals were primarily localized in the cytosol (Supplementary Fig. 28). Along with the disintegration of particles, GSH within cancer cells was depleted to varying degrees, depending on the internalization efficiency of samples (Supplementary Fig. 29). However, GSH levels in MCF-10A and HC11 cells were consistently low.

Recent reports have confirmed that cancer cell killing by PPE is dependent on its proteolytic liberation of CD95 C-terminal fragment DD. Therefore, we first determined if PPE loaded on the nanodevice could cleave CD95 in cells. Western blotting results showed that FKPN and FPN promoted significantly higher liberation of CD95 DD in cancer cells than FKN (Supplementary Fig. 30a, b). While in normal cells, FKPN failed to free intracellular DD of CD95. And the histone H1 isoforms (H1.0) were indeed elevated in cancer versus normal cell lines (Supplementary Fig. 30c). 2,7-dichlorofluorescein diacetate (DCFH-DA) detector was used to probe the photo-induced ^1^O_2_ production. Flow cytometry analysis showed that FKPN effectively upregulated the levels of ^1^O_2_ within MDA-MB-231 and 4T1 cells instead of MCF-10A and HC11 cells upon laser irradiation (Fig. 3d). 3D-CLSM images further confirmed that for cancer cells, FKPN and FKN groups exhibited much stronger nuclear ^1^O_2_ signals than FPN group under laser irradiation (Fig. 3e and Supplementary Fig. 31). For MCF-10A and HC11 cells treated with FKPN with laser irradiation, they showed very feeble ^1^O_2_ signals throughout the entire cell, which was comparable to that of PBS group. Then, the amount of DNA DSBs induced by PPE and ^1^O_2_ was evaluated by western blotting and neutral comet assay. Compared with FKN and FPN groups upon light irradiation, FKPN obviously increased phosphorylation of γH2AX (a DSB marker positively correlated with early transformation and genomic instability) and the ratio of γH2AX/H2AX within cancer cells (Fig. 3f and Supplementary Fig. 32). Negligible phosphorylation was observed in MCF-10A and HC11 and blank control groups. Comet assay results showed that comet tails in cancer cells were significantly longer in FKPN plus irradiation group relative to other groups (Fig. 3g and Supplementary Fig. 33). These data indicated that FKPN selectively induced severe DSBs in cancer cells by combining cancer cell-specific nuclear ^1^O_2_ generation and PPE. Subsequently, immunofluorescence assays showed that FKPN plus laser irradiation triggered significantly more cytoplasmic histone H1.0 localization and CD95 DD-H1.0 interactions in MDA-MB-231 and 4T1 cells, but not in MCF-10A and HC11 cells (Fig. 3h and Supplementary Fig. 34). High colocalization of CD95 DD-H1.0 with mitochondria (Mito) was also observed in cancer cells treated with FKPN plus laser irradiation (Supplementary Fig. 35). Co-immunoprecipitation (coIP) assays reconfirmed that FKPN induced increased binding of CD95 DD to H1.0 within MDA-MB-231 and 4T1 cells under laser irradiation (Fig. 3i and Supplementary Fig. 36). The above data indicated that just in cancer cells, enhanced histone H1 translocation in response to PPE and nuclear ^1^O_2_ generation induced more bindings of CD95 DD and histone H1.0 isoform.

### Precision discrimination and eradication of cancer cells by FKPN

**Fig. 4:**
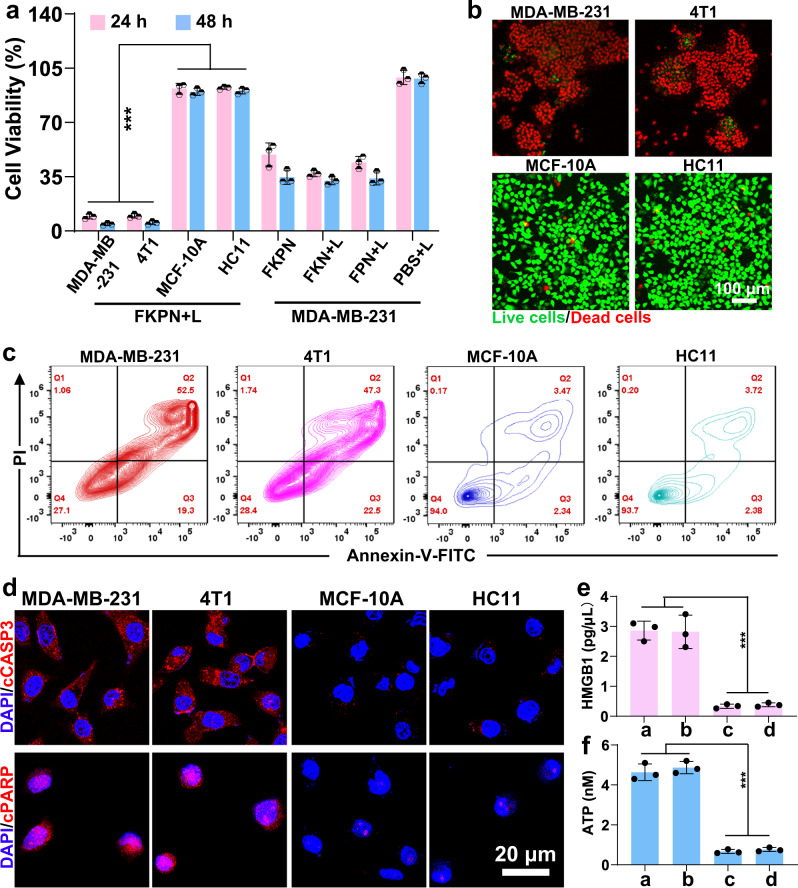
FKPN combined with laser irradiation induces ICD of cancer cells. **a** CCK-8 assay of cancer and non-cancer cell viability after culture with FKPN for various times and treated with laser irradiation. Data were presented as mean ± SD (*n* = 3 independent experiments). *P* values: 2.34443E-07. **b** CLSM images of live/dead staining of treated cells. **c** Flow cytometry analysis of the apoptosis of cancer and non-cancer cells after treatment with FKPN plus laser irradiation. **d** Apoptosis pathway analysis of treated cancer and non-cancer cells assessed by immunofluorescence staining of cCASP3, and cPARP. **e** Quantification of released HMGB1 from cancer and non-cancer cells. Data were presented as mean ± SD (*n* = 3 independent experiments). *P* values: 0.000436634. **f** Amount of released ATP from cancer and non-cancer cells after treatments. **a**: MDA-MB-231; **b**: 4T1; **c**: MCF-10A; **d**: HC11. Data were presented as mean ± SD (*n* = 3 independent experiments). *P* values: 3.99556E-06. P values were assessed using Student’s *t* test (two-tailed) (***p < 0.001). **b**–**d** Representative of *n* = 3 independent experiments. Source data are provided as a Source Data file.

We next evaluated the targeted killing effect of nanodevice. The CCK-8 assay revealed that FKPN showed obvious cell proliferation inhibition toward cancer cell line, with approximately 95.8% and 94.8% cell death upon irradiation (Fig. 4a), and exhibited negligible effect on the non-cancer cell survival. Live/dead staining assay further verified the coexistence of FKPN and irradiation led to more cell death in cancer versus non-cancer cells (Fig. 4b and Supplementary Fig. 37a). Flow cytometry analysis suggested that FKPN plus irradiation treatment promoted the apoptosis of cancer cells, but did not cause evident apoptosis in non-cancer cells (Fig. 4c and Supplementary Fig. 37b). Furthermore, the mechanism of cancer cell apoptosis induced by FKPN plus irradiation was investigated. The highest level of PARP and CASP3 cleavage (cPARP and cCASP3) in FKPN plus irradiation group suggested that CD95 DD-H1.0 co-localized in mitochondria, inducing mitochondrial damage and initiating apoptotic MDA-MB-231 cell death (Fig. 4d and Supplementary Figs. 38, 39). In contrast, this was not observed in MCF-10A and HC11 cells. Of note, both PPE and ROS were previously reported to trigger immunogenic cell death (ICD) for immune activation^53,54^, so we studied the induction of ICD effect by FKPN. As expected, FKPN plus laser irradiation treatment induced obviously enhanced high-mobility group box 1 (HMGB1) release in cancer cells (Fig. 4e and Supplementary Fig. 40a). Furthermore, an overwhelming increase of adenosine triphosphate (ATP) secretion (hallmark for ICD) was observed in FKPN plus laser irradiation-treated MDA-MB-231 and 4T1 cells (Fig. 4f and Supplementary Fig. 40b). These data indicated that FKPN efficiently induces ICD of cancer cells upon irradiation. Collectively, the above results indicated that the FKNP could realize precise effective killing to target cancer cells under laser irradiation by combining innate immune factor PPE and tumor-specific histone H1 translocation.

### In vivo immune activation and antitumor effect of FKPN

**Fig. 5:**
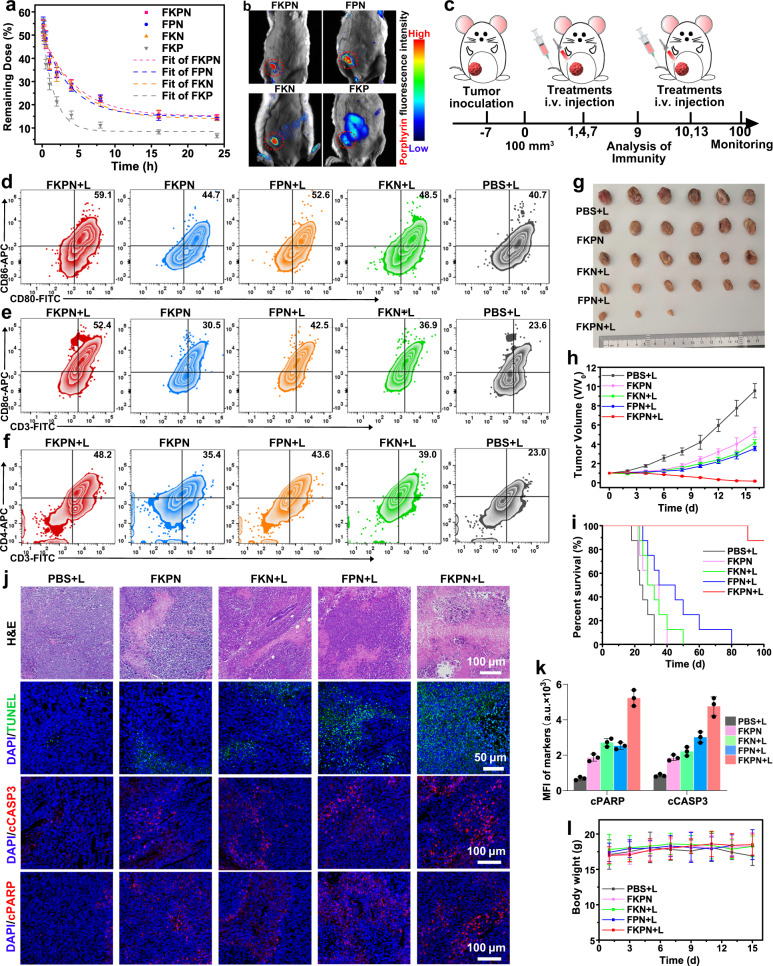
In vivo immune activation and antitumor effect of FKPN plus laser irradiation. **a** In vivo pharmacokinetic curves within 24 h after intravenous injection of samples. Data were presented as mean ± SD (*n* = 3 mice). **b** In vivo fluorescence images of tumor-bearing mice after being injected with different particles for 3 days. Red: porphyrin fluorescent. **c** Schematic illustration of treatment schedule using a 4T1 orthotopic breast cancer BALB/c mouse model. **d** Flow cytometry analysis of DC maturation in lymph nodes on day 9. Flow cytometric analysis of tumor-infiltrating **e** CD8^+^ T cells and **f** CD4^+^ T cells after various treatments. **g** Representative photographs of the tumors excised from tumor-bearing mice on day 16. (*n* = 6 mice). **h** Relative tumor volume change of 4T1 tumor models during the therapy for 16 days. Data were presented as mean ± SD (*n* = 6 mice). **i** The survival percentages of treated mice. (*n* = 8 mice). **j** H&E or TUNEL immunofluorescence staining of tumor tissue from different groups after 16 days; representative immunofluorescence images of cCASP3 and cPARP staining on tumors after the final treatment. **k** Quantification of cCASP3 and cPARP signals in tumor sections according to **j** Data were presented as mean ± SD (*n* = 3 mice). **l** Body weight of each group of mice during various treatments. Data were presented as mean ± SD (*n* = 6 mice). **b**, **d**, **j** Representative of *n* = 3 independent experiments. Source data are provided as a Source Data file.

Encouraged by the in vitro selective killing of cancer cells, we further explored the therapeutic efficacy of FKPN in immunocompetent mice bearing 4T1 tumors. The biosecurity of FKPN was first assessed. Hematological analysis results showed that blood biochemical and physiological parameters of healthy BALB/c were all in the normal range 5 d after intravenously (*i.v*.) administration (Supplementary Fig. 41a). Moreover, no noticeable weight loss was observed in FKPN group during the three-week evaluation period (Supplementary Fig. 41b). Hematoxylin-eosin (H&E) staining of main organs displayed negligible tissue abnormality (Supplementary Fig. 41c). These findings suggested good biosafety of the samples. The blood circulation half-life of FKPN (*t*_1/2_ = 4.98 h), FPN (*t*_1/2_ = 4.06 h) and FKN (*t*_1/2_ = 4.51 h) was significantly longer than that of FKP (*t*_1/2_ = 1.43 h), suggesting the superior stealth property of NM camouflage (Fig. 5a and Supplementary Fig. 42). Given that the prolonged circulation time and retention of representative adhesion proteins on membrane coating, we investigated whether the FKPN had tumor targeting and accumulation capability. In vivo fluorescence imaging results showed that strong red fluorescence signals of FKPN, FKN, and FPN were detected in tumor regions 4 h after *i.v*. injection, and reached maximum at 24 h after administration (Fig. 5b and Supplementary Fig. 43a). Significantly, intense fluorescence was still observed up to 72 after injection. Ex vivo fluorescence imaging of harvested tissues also showed over 12-fold tumor accumulation of NM-camouflaged NPs compared to that of NP without NM coating (Supplementary Fig. 43b, c). Together, these results confirmed the preferential tumor targeting and accumulation of FKPN. Notably, the fluorescence signals could not only track tumors but also monitor nanodevice decomposition to promote failsafe and efficient cancer treatment.

Next, the in vivo immune activation was investigated since our in vitro studies had shown that FKPN could induce ICD. After three consecutive times treatments, the mice were sacrificed to extract cell populations from tumor-draining lymph nodes and tumors for further immune analysis (Fig. 5c). Flow cytometry analysis indicated that all treatment groups promoted dendritic cell (DC) maturation (CD80^+^CD86^+^) to some extent compared with the PBS group, and FKPN plus laser irradiation exhibited an elevated level of mature DCs with a percentage of 60.0% (Fig. 5d and Supplementary Fig. 44a). Consistently, upon laser irradiation, FKPN treatment exhibited a higher frequency of CD8^+^ T cells compared with controls (Fig. 5e and Supplementary Fig. 44b). Notably, FKPN plus irradiation induced the highest percentage of CD4^+^ T cells to 48.2% in tumors (Fig. 5f and Supplementary Fig. 44c). In addition, the effector memory T cells (CD44^+^CD62L^-^CD4^+^ and CD44^+^CD62L^-^CD8^+^) in the spleen were detected using flow cytometry analysis. Treatment with FKPN plus irradiation resulted in a significant expansion of these two T-cell subsets with effector memory phenotypes compared with the control (Supplementary Fig. 45). All these results indicated that FKPN plus laser irradiation induced a robust immune response in tumor tissue through PPE combined with Histone H1 translocation-mediated cell death mechanism. Consequently, after treated by FKPN plus irradiation, 50% tumors were eradicated (Fig. 5g, h), and noticeable pathological alterations of tumor slices and the highest survival benefit was achieved (Fig. 5j, i). Furthermore, immunofluorescence staining showed the strongest increase in TUNEL, cPARP and cCASP3 (cleaved PARP and CASP3) signals in in the FKPN plus irradiation treatment group (Fig. 5j, k), indicating elevated DNA damage and apoptosis. More importantly, body weight changes of mice and H&E analyses of major organs of all groups confirmed the reliable biosecurity of our nanodevice (Fig. 5l and Supplementary Fig. 46). To further investigate the establishment of immune memory, we then rechallenged cured mice from the FKPN plus irradiation treatment group by subcutaneously inoculating 4T1 cells on the opposite flank. Tumor growth in rechallenged mice was significantly inhibited, while it showed predictable kinetics in age-matched, treatment-naïve mice (Supplementary Fig. 47). Together, these findings proved that FKPN plus irradiation could effectively achieved antitumor therapy by inducing tumor apoptosis and activating a robust antitumor immunity.

### FKPN induces systemic immune response-mediated abscopal effect

**Fig. 6:**
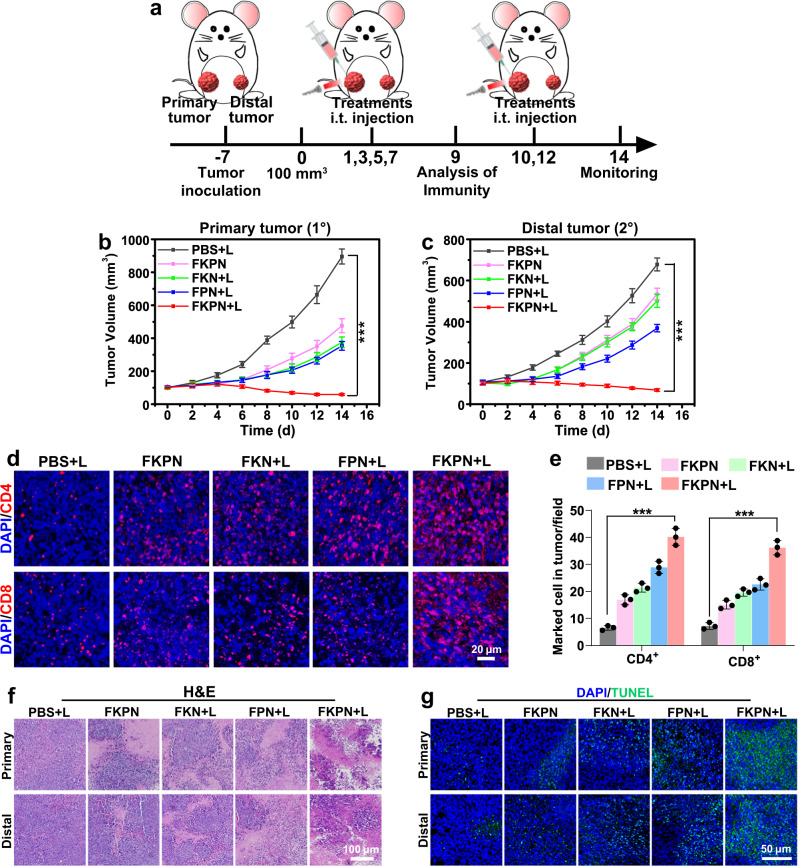
FKPN combined with laser irradiation induces systemic immune response-mediated abscopal effect. **a** Schematic illustration of treatment schedule using bilateral 4T1 breast cancer BALB/c mouse model. **b** Primary and **c** distal tumor growth curves of mice after various treatments. Data were presented as mean ± SD (*n* = 6 mice). *P* values: 7.25826E-13 for primary tumor volume of mice in FKPN + L group vs in PBS + L group on day 14; 4.3309E-13 for distant tumor volume of mice in FKPN + L group vs in PBS + L group on day 14. **d** Immunofluorescence images of distal tumor tissues stained for CD4^+^ and CD8^+^ on day 9. **e** Quantification of CD4^+^ and CD8^+^ signals in tumor sections according to **d**. Data were presented as mean ± SD (*n* = 3 mice). *P* values: 5.75306E-05 for CD4^+^ signals in FKPN + L group vs in PBS + L group; 6.65982E-05 for CD8^+^ signals in FKPN + L group vs in PBS + L group. **f** H&E staining and **g** TUNEL staining of the primary and corresponding distant tumor section after various treatments. *P* values were assessed using Student’s *t* test (two-tailed) (***p < 0.001). Source data are provided as a Source Data file.

We further evaluated whether treating a primary tumor with FKPN plus irradiation could trigger systemic immune response capable of delaying distant tumor growth, a property termed as the abscopal effect (Fig. 6a). As expected, the combination of FKPN with laser irradiation significantly attenuated primary tumor growth, in comparison with other treatment groups (Fig. 6b, c and Supplementary Fig. 48). Meanwhile, an abscopal effect was present on mice of FKPN plus irradiation group, where the untreated distant tumor size was suppressed by 90% compared with that in PBS group. This abscopal effect also possessed tumor specificity, since treating primary tumor with FKPN plus laser irradiation did not retard tumor growth in a genetically distinct distant B16F10 tumor (Supplementary Fig. 49). Considering the above demonstrated mechanism by which FKPN activates the immune system, we deduced that treating a primary tumor with FKPN plus irradiation could mediate abscopal effect by boosting specific T-cell activation and infiltrating. The results from immunofluorescence staining suggested that the frequency of CD4^+^ T helper cells infiltrating into distant tumor in the FKPN plus irradiation group was significantly increased (Fig. 6d, e). Meanwhile, cytotoxic CD8^+^ T cells in distal tumors of FKPN plus irradiation group remained at a highest level. The enhanced tumor cell killing was confirmed by obvious apoptosis and necrosis morphology in primary and distant tumor (Fig. 6f, g). Similarly, no abnormalities were observed in major organs from each group, indicating that the treatment had caused little systemic toxicity under our experimental conditions (Supplementary Fig. 50). We also performed DC and T-cell blocking experiments to further confirm the dependence of the efficient abscopal effect on these immune cells (Supplementary Figs. 51 and 52a, b). 4T1 bilateral breast cancer mice depleted of DCs, CD4^+^, or CD8^+^ T cells were treated with FKPN and laser irradiation as described above. Results showed that primary tumor inhibition was attenuated and the abscopal effect was abolished in the case of depletion of either DC or T cells, whereas it was not affected in Mouse IgG control group (Supplementary Fig. 52c, d). These results suggested that DCs, CD8^+^, and CD4^+^ T cells were required not only for the abscopal effect but also for primary tumor suppression.

## Discussion

We design and develop a tumor discrimination nanodevice to precisely treat solid tumors independent of antigen recognition. Due to heterogeneity and limited tumor specificity of antigen in a solid tumor, development of tactics based on the other mechanisms of tumor selectivity is highly desirable. Our constructed FKPN can target solid tumor site and specifically unlock in cancer cells in response to high GSH. The released PPE and porphyrin-NLS initiate tumor cell killing based on overexpressed histone H1 isoform, which facilitates further tumor identification. PPE proteolytically liberating the CD95 DD, and CD95 DD interacts with histone H1 in cytosol, simulating neutrophils’ mechanism of action. Porphyrin grafted with NLS can enter the nucleus and generates ^1^O_2_ in-situ under laser irradiation, promoting histone H1 nucleo-cytoplasmic translocation by inducing DSBs. The resulting elevated levels of CD95 DD-H1.0 interactions in cancer versus normal cells will induce cancer cell apoptosis with little toxicity to normal cells. In vivo studies demonstrate that FKPN eliminates primary tumor growth, promotes DC maturation and elicits an adaptive T-cell response-mediated abscopal effect, thereby attenuating distal tumor growth. Given that PPE is currently being developed for clinical use and all the findings in this work, our design provides a promising strategy for clinical solid tumor therapy.

## Methods

### Cell lines and mice

MCF-10A cell line (Catalog no. FH0220) and HC11 cell line (Catalog no. FH1191) were purchased from and authenticated by Fuheng Biology. MDA-MB-231 cell line (Catalog no. HTB-26) and 4T1 cell line (Catalog no. CRL-2539) were purchased from and authenticated by ATCC. These cell lines were tested negative for mycoplasma contamination by the suppliers. All cell lines in this work are not listed by International Cell Line Authentication Committee as cross-contaminated or misidentified cell lines (v12, 2023).

190 BALB/c mice (6-week old) of both sexes were obtained from Medical Experimental Animal Center of Jilin University (Changchun, China). The handling procedures were in compliance with the guidelines of Jilin University Animal Care and Use Committee. Mice were maintained at 12-h light-dark cycle within 22–26 °C and 40–60% humidity. The maximal tumor burden permitted by the protocol of the Animal Care and Use Committee of Jilin University did not exceed 2000 mm^3^. In our work, the maximal tumor size was not exceeded.

### Synthesis of Fe-porphyrin MOF

The Fe-porphyrin MOF was prepared via a solvent-thermal method. In brief, FeCl_3_·6H_2_O solution (2 mL, 10 mg/mL in DMF), H_2_TCPP solution (4 mL, 2.5 mg/mL in DMF), and benzoic acid solution (4 mL, 100 mg/mL in DMF) were sequentially added into a flask, followed by stirring at 90 °C for 5 h. The mixture was cooled down, respectively washed three times by DMF and ethanol, and then dried at 60 °C for 5 h.

### Synthesis of NLS-grafted Fe-porphyrin MOF (FK)

The Fe-porphyrin MOFs were dispersed in DMSO (1 mg/mL, 10 mL) by ultrasonic bath for 30 min and then 2 mL MES buffer (pH 6.0, 20 mM) containing EDC (3 mg) and NHS (2 mg) was added. Then, this mixture was incubated with vigorous shaking overnight in the dark and subsequently added 1.0 mM KRRRRG solution (diluted in Milli-Q water, 2 mL). After shaking for 24 h, the obtained production was washed and then redispersed in 50% TFA solution diluted in DCM (30 mL). This mixture was stirred for 4 h, followed by washing with Milli-Q water for further use.

### Peripheral neutrophil collection and activation

The Percoll gradient method was used to isolate neutrophils from the BALB/c mice^40^. Briefly, the peripheral blood of mice was collected, purified by centrifugation (700 × *g*, 10 min) at 4 °C, and subsequently resuspended in PBS with EDTA. The obtained suspension was slowly added to the top of a three-layer percoll gradient of 78%, 65%, and 55%, and then centrifuged (1500 × *g*, 30 min). As a result, blood cells were layered and neutrophils were collected from the interface of the 65% and 78% fractions, and washed twice with ice-cold PBS. After lysing erythrocytes by lysis buffer, purified neutrophils were obtained. LPS (100 ng/mL) was added to the isolated neutrophil-culture medium. Neutrophils were further cultured for 4 h in an incubator to obtain activated neutrophils, followed by washing with PBS.

### Neutrophil membrane preparation

After washing with cold PBS, the activated neutrophils were then resuspended in hypotonic lysing buffer containing protease inhibitor. The neutrophils were disrupted with a homogenizer to obtain the suspension. After centrifugation (10,000 × *g*, 30 min) at 4 °C, the supernatant was obtained. Then the sample was centrifuged again (100,000 × *g*, 30 min, 4 °C). The obtained membrane material was washed using 0.2 mM EDTA in water containing protease inhibitor, followed by freeze-drying, weighting, and storing at −80 °C.

### Synthesis of biomimetic nanodevice equipped with porcine pancreatic elastase (PPE) and neutrophil membrane (NM) coating (FKPN)

The FK particle dispersion (5 mg/mL, 2 mL) was added dropwise into PPE solution (20 μM in water, 10 mL) with the help of stirring and maintained for 5 h. The supernatants were collected for BCA protein assay (Absin, Shanghai, China) to determine the protein loading efficiency. The obtained precipitate (FKP) was collected for the following reaction. Thermogravimetric analyses (TGA) were performed with a Pyres 1 TGA apparatus (Perkin Elmer, MA) with a heating rate of 10 °C/min under an air atmosphere (from 50 °C to 800 °C).

For membrane coating, NM was mixed with FKP cores at a weight ratio of membrane: core = 1:2. After sonicated (100 w, 1 min, sonication for 2 s, intervals for 3 s) in an ice-bath, this mixture was repeatedly extruded through 400 nm polycarbonate porous membranes. The final FKPN NPs were obtained by centrifugation (10,000 × *g*, 20 min) at 4 °C.

For control, the particles without PPE loading (FKN) or without NLS grafting (FPN) were synthesized. 2 mL FK (water solution, 1 mg/mL,) was added into 1 mL NM (1 mg/mL in water). The obtained mixture was sonicated on an ice-bath as mentioned above. After centrifugated at 10,000 × *g* for 20 min, the FKN was obtained. For FPN, 10 µL of PEI was added into Fe-porphyrin MOF solution (2 mg, 10 ml) and stirred for 10 h at room temperature. After centrifugation and washing, the sample was subjected to PPE loading and NM coating process according to procedure described above.

Furthermore, the PPE and NM were conjugated with Rhodamine B (RhB) and Rhodamine 110 (Rh110) respectively to monitor the loading and coating of FKPN by confocal microscope (Nikon Eclipse Ni-E, Japan) observation.

### Characterization of membrane-associated protein

Protein characterization was carried out using Coomassie blue staining and western blot. Briefly, 10 µL of membrane fragments, the FKPN and FKP NPs were loaded into the well of sodium dodecyl sulfate-polyacrylamide (SDS-PAGE) gels. Coomassie Blue combined with DNR Bio-imaging Systems (MicroChemi) was used to image total protein. For western blotting, the proteins were transferred to an activated PVDF membranes (Millipore) and probed with corresponding primary antibodies and horseradish peroxidase (HRP)-conjugated secondary antibodies (Wuhan Fine Biotech Co., Ltd.). Detailed information of antibodies was given in Supplementary Table 1. The films were imaged using enhanced chemiluminescence detection (ECL-Plus kit, Beyotime, China) and Tanon 4800 Automatic Chemiluminescence Image Analysis System.

#### Characterization of GSH-triggered FKPN disintegration

The disintegration of FKPN in response to GSH was examined by analyzing the chemical states of iron using a Perkin Elmer PHI 5600 XPS. TEM imaging was used to record the morphology change of FKPN. Furthermore, the GSH depletion was measured using Ellman’s reagent^50^. Briefly, DMSO solution containing DTNB (5 μL, 100 mM) was mixed with GSH solution (995 μL) with different concentrations (0, 0.625, 1.25, 2.5, 5, 10 mM). The absorbance of solutions in 24-well plates were determined by a Bio-Rad model-680 microplate reader (USA) at λ = 405 nm after incubation for 2 min, establishing A_GSH_ standard curve. Meanwhile, the standard curve of nanoparticles (NPs) was determined through measuring the absorbance of NP solution (420 nm). Then different concentrations of GSH solutions (C_0_, 995 μL) were incubated with a certain concentration of NPs, followed by adding 5 μL of DMSO containing DTNB (100 mM). The absorption of the solutions in 96 well plates was detected by microplate reader at *λ* = 405 nm after standing for 10 min. The background absorption was subtracted from the sample absorption. The depletion of GSH (C_X_) were calculated by the formula: C_X_ = (A_X_ − A_NPs_)/(A_GSH_ − A_NPs_) × C_0_.

### Evaluation of cargo release from NPs

To determine GSH-triggered porphyrin release, 10 μL of NP solution was mixed with 990 μL of GSH solutions with different concentrations (0, 0.625, 1.25, 2.5, 5, and 10 mM) at 37 °C. The fluorescence signal of porphyrin in the mixtures was detected using a JASCO F-6000 fluorescence spectrometer (Ex = 420 nm, Em = 630 nm) after incubation for 20 min. Fe content in the supernatant was detected via inductively coupled plasma mass spectrometry (ICP-MS). To study the release kinetics of PPE from NPs, the complex was incubated in PBS buffer containing or not containing GSH (2.5 mM) at 37 °C for 8 h. At different time points, a certain amount of solution (1 mL) was taken out and centrifuged (10,000 × *g*, 10 min). The BCA protein assay (PPE as the standard protein) was used to quantify the PPE concentration in supernatant.

#### PPE activity assays

The catalytic activity of released PPE was measured using the substrate pNA (100 mg/mL)^27^. Along with supernatant obtained in the above procedure, the substrate was added into a well of a 96 well plate in a final concentration of 100 μg/mL and in a total volume of 100 μL/well. The absorption at 405 nm was detected by a microplate reader. The background absorbance was subtracted from the sample absorbance. One unit was defined as the amount of PPE that hydrolyzed 1 nmol of substrate per min at 37 °C.

### Singlet oxygen (^1^O_2_) detection

SOSG agent was employed to monitor ^1^O_2_ generation. Adding 10 µL of SOSG solution (5 µM) into 990 μL NP solution (10 μg/mL) containing different concentrations of GSH. After 4 min of laser irradiation (0.3 W/cm^2^ at 660 nm), the fluorescence signal of solution at 550 nm was detected at different time points.

#### Cell culture

MDA-MB-231 cells were grown on plates with DMEM medium containing 10% FBS, penicillin (100 μg/mL), and streptomycin (100 μg/mL). MCF-10A cells were cultured in DMEM/F12 medium containing 10% FBS, penicillin (100 μg/mL), and streptomycin (100 μg/mL). 4T1 cells and HC11 cells were cultured in RPMI 1640 medium with 10% FBS, penicillin (100 μg/mL), and streptomycin (100 μg/mL). All cells were maintained in an incubator containing 5% CO_2_ at 37 °C. The medium was changed every other day.

#### Cell uptake

Various cells were cultured onto a 24-well plate (NEST Biotechnology Co. Ltd., Wuxi, China) and allowed to grow to 80% confluency. After that, the cells were co-incubated with different nanoparticles for 4 h, followed by washing with PBS. After fixed by 4% polyformaldehyde, the cells were incubated with DAPI (1:1000, Sigma-Aldrich) for 15 min and observed by CLSM.

#### Analysis of adhesion proteins on cells

MDA-MB-231 cells and 4T1 cells were cultured onto a plate and allowed to grow to 80% confluency. Then the cells were trypsinized, washed with PBS and incubated with anti-CD44-FITC and anti-ICAM-1-FITC antibodies, respectively for 30 min at 4 °C. After washing with PBS, the protein expression in cells were detected by flow cytometry. To perform adhesion protein blockade, both cell types were preincubated with anti-CD44 or anti-ICAM-1 antibodies for 2 h at a dose of 2 mg/mL prior to flow cytometry analysis. The FACS gating strategies are shown in Supplementary Fig. 53b.

#### Uptake mechanism of FKPN NPs

To evaluate the uptake mechanism of for FKPN NPs, MDA-MB-231 cells and 4T1 cells were pretreated with various inhibitors for 2 h at 37 °C: chlorpromazine (clathrin-mediated endocytosis inhibitor, 10 μg/mL), genistein (caveolin-mediated endocytosis inhibitor, 200 μM), cytochalasin D (macropinocytosis inhibitor, 2.5 μg/mL) and nystatin (inhibitor of lipid raft-caveolae endocytosis, 2.5 µg/mL). In addition, the cells were cultured at 4 °C to assess the effect of temperature on FKPN uptake. After that, the cells were cultured with fresh medium containing inhibitors at the same concentrations and FITC-labeled FKPN for 4 h. After washing with PBS for three times, the uptake of the FKPN by cells were analyzed through flow cytometry. The cells without any treatment were used as the background during the flow cytometry analysis, while the cells treated with of FKPN NPs only were served as the control. The FACS gating strategies are shown in Supplementary Fig. 53c.

For colocalization assay, both cancer cells were co-incubated with FKPN NPs for 1, 2, and 4 h, respectively. After washing by PBS, both cancer cells were co-incubated with lysosomal indicator LysoTracker red (1:15000, Beyotime) for 30 min, followed by staining with Hoechst 33342 (1:1000, Sigma-Aldrich). The cells were imaged by CLSM and the fluorescence intensity was determined by Image J V1.8.0. software.

#### Analysis of intracellular NP disintegration

Various cells were cultured on a plate and allowed to grow to 80% confluency. After that, different samples were added to the wells, followed by 4 h incubation. Next, the cells were washed by PBS, fixed by 4% polyformaldehyde, and co-incubated with DAPI for 15 min before fluorescence analysis. The labeled cells were imaged using CLSM. Moreover, PPE (10 mg) and FITC (10 mg) were dispersed in aqueous solution (20 mL) with moderate stirring for 12 h to obtain FITC-labeled PPE to study the uptake of protein by cells.

#### Evaluation of cellular GSH levels

Various cells were cultures onto a 24-well plate and allowed to grow to 80% confluency. After that, all cells were treated by different particles at an equivalent dose of 20 µg/mL porphyrin for 4 h. After removing the medium, 100 μL of lysis buffer was added in wells to lyse the cells. The lysates were centrifuged (10,000 × *g*, 10 min), and then the obtained supernatant (50 μL) was mixed with 250 μL DTNB solution (0.5 mM). The GSH concentration was determined by detecting the absorption of mixture at 405 nm. The background absorbance was subtracted from the sample absorbance.

#### Western blot-based analysis of liberation of CD95 death domain (DD)

To perform western blot assay, cells were harvested after 6 h in culture in medium containing different samples. Then cells were lysed in chilled RIPA lysis (Life-iLab, Shanghai, China) containing protease inhibitors, and the total protein was quantified by BCA protein assay. The proteins resolved on 12% SDS-PAGE gel and transferred to PVDF membranes, which were subsequently blocked with 5% nonfat milk (4 °C, 12 h). After incubating with primary and secondary antibodies, the membranes were visualized by ECL western blotting substrate (BestBio) and Tanon 4800 Automatic Chemiluminescence Image Analysis System.

#### Western blot-based analysis of cellular histone H1.0 levels

Various cells were allowed to grow to 90% confluency. Subsequently, all cells were lysed and the mixtures were performed western blotting analysis according to protocols mentioned above.

#### Intracellular ^1^O_2_ detection

DCFH-DA served as a capture probe to detect intracellular ^1^O_2_ generation. Various cells were plated in a 24-well microplate and grown to 80% confluency. Then different samples were added into the cells at an equivalent dose of 20 µg/mL porphyrin. After 4 h incubation, the cells were co-incubated with DCFH-DA (10 μM) for 15 min, suspended in fresh PBS and irradiated with 660 nm laser (0.3 W/cm^2^, 3 min). The fluorescence signal was detected by flow cytometry or were directly observed by CLSM. The FACS gating strategies are shown in Supplementary Fig. 53a.

#### Detection of DNA damage by western blot and comet assay

Various cell lines were plated on a 24-well microplate and grown to 80% confluency. After incubation with different samples for 4 h, several groups of cells were treated with 660 nm laser (0.3 W/cm^2^, 3 min). All cells were further cultured for 4 h, followed by lysing and immunoblotting for γH2AX protein levels as described above.

For comet assay, the treated cells were digested, washed, and resuspended in PBS. 50 μL of cell suspension was taken out and mixed with 150 μL low-melting agarose (LMA, 0.5%). Afterward, cell-agarose mixture was spread on comet slides precoated with normal-melting agarose (NMA, 0.5%), and the slides were incubated at 4 °C for 3 h. Subsequently, the slides were placed in precooled neutral lysis buffer, unwound in the chilled neutral electrophoresis buffer, and subjected to electrophoresis. After the slides being neutralized and air dried, they were stained with the DAPI in the dark for 20 min and observed with CLSM.

#### Immunofluorescent staining-based analysis of cytoplasmic histone H1.0 localization, CD95 DD-H1.0 interaction, and mitochondrial trafficking of H1.0

After incubation with different formulations for 4 h, the cells were treated with or without laser (0.3 W/cm^2^, 660 nm) for 3 min, and cultured for another 4 h. After fixing with 4% (w/v) paraformaldehyde for 10 min, the cells were washed and permeabilized by 0.1% Triton-X before blocking with bovine serum albumin (BSA, 5%) solution for 1 h. Then the cells were labeled with primary antibodies and secondary antibodies. For mitochondrial labeling, the treated cells were incubated with Mito-Tracker Green (200 nM) for 30 min after antibody labeling. The nuclei of the cells were counterstained by incubating the cells with DAPI at 25 °C for 15 min prior to imaging via CLSM under identical exposure conditions for all the groups compared. Furthermore, the fluorescence signal was analyzed via Image J software.

#### Co-immunoprecipitation (co-IP) experiment-based analysis of H1.0 interaction with CD95 DD

Co-IP analysis was performed according to standard procedures. In detail, cells were plated on a 6-well microplate and grown to 80% confluency. After 4 h incubation with different formulations, the cells were treated with or without laser (0.3 W/cm^2^, 660 nm) for 3 min, and further cultured for 4 h. The treated cells from all groups were cultured with RIPA buffer containing fresh phosphatase inhibitor and protease inhibitor. The lysates were incubated with Flag-conjugated agarose beads (30 µl, Proteintech) at 4 °C for 12 h. Affinity-bound protein-agarose beads were washed and eluted with non-reducing sample buffer. Then, western blot assays of proteins were performed with corresponding antibodies.

#### Evaluation of selective cancer cell killing

Cell viability was evaluated by CCK-8 kit (Scilia) and live/dead co-stained assay. Various cells were co-incubated with different formulations for 48 h, followed by washing with PBS and incubating with 100 μL of CCK-8 solution for 1 h. Absorbance of the supernatant of each group in 96 well plate was measured by microplate reader at *λ* = 450 nm. The background absorbance was subtracted from the sample absorbance. For live/dead cell analysis, the treated cells from all groups was washed with PBS, labeled with calcein-AM (green) and propidium iodide (red) for 30 min at 37 °C, and then observed by CLSM. The live and dead cells were counted to determine viability of the cells.

Flow cytometry assay was performed to evaluate the cell apoptosis. After being treated with different formulations, the cells in all groups were washed, digested with trypsin, and labeled with Annexin V-FITC Apoptosis Detection Kit (Beijing T&L Biological Technology Co., Ltd), followed by flow cytometry analysis. The FACS gating strategies are shown in Supplementary Fig. 54.

#### Detection of ICD marker ATP and HMGB1

The amount of extracellular released ATP was quantified by an ATP assay kit (Beijing Solarbio Science & Technology Co., Ltd, BC0300). After co-incubated with different samples for 4 h, the cells were treated with or without laser (660 nm, 0.3 W/cm^2^) for 3 min, and further cultured for 4 h. The ATP level in culture medium were detected according to the manufacturer’s protocols.

ELISA assays were carried out to determine the level of HMGB1 in different cell lines. Similarly, cells were co-incubated with different samples for 4 h, followed by treating with or without laser (660 nm, 0.3 W/cm^2^) for 3 min and culturing for another 4 h. The release of HMGB1 in the cell medium was detected by the HMGB-1 ELISA Kit (Shanghai hengyuan biological technology co., LTD, B163318 and HY-10607K) according to the manufacturer’s protocols.

#### In vivo biocompatibility evaluation

Healthy BALB/c mice were randomized based on sex, age, and weight, with three mice in each group. PBS and FKPN (NP concentration: 10 mg/kg, 50 μL) were i.v. injected into the mice, respectively. Body weights of mice were recorded during administration period (21 days). At 5 d after injection, several mice of each group were sacrificed and the blood of mice was collected for biochemical and hematologic analysis. Finally, H&E analyses of major organs were performed to evaluate tissue toxicity of FKPN.

#### Plasma pharmacokinetic analysis

To access NP pharmacokinetics, the healthy BALB/c mice were i.v. administered with different nanoparticles (10 mg/kg, with equal iron). The mice were randomized based on sex, age, and weight, with three mice in each group. 30 µL blood samples were repeatedly collected by tail snip at 0, 0.25, 0.5, 1, 2, 4, 8, 16, and 24 h postinjection and analyzed using ICP-MS (Varian 720-ES).

#### In vivo and ex vivo biodistribution imaging

First, 4T1 cells (1 × 10^6^) were injected into the mammary gland of BALB/c mice to set up xenograft tumor model. The mice were randomized based on sex, age, and weight, with 3 mice in each group. Once the tumor volume reached 150 mm^3^ (0.5 × [long axis] × [short axis]^2^), 50 μL of FKPN, FKN, FPN, and FKP in PBS were *i.v*. injected (10 mg/kg). At 1, 2, 4, 8, 12, 24, 48 and 72 h after injection, the mice were visualized by AniView600 in vivo fluorescent imaging system (Biolight, Guangzhou, China). At 72 h post i.v. injection, various tissues (tumor and organs) of mice were collected and imaged for biodistribution study.

#### In vivo immune activation analysis and antitumor study

BALB/c mice bearing 4T1 xenograft tumor were divided into 5 groups with 20 mice in each group: PBS, FPN + irradiation, FKN + irradiation, FKPN or FKPN + irradiation (nanoparticles: 10 mg/kg, laser irradiation: 0.3 W/cm^2^ and 5 min). The mice were randomized based on sex, age, and weight. The treatments were conducted every three days once tumor reached about 100 mm^3^ (day 0). Of the 20 mice in each group, 6 mice were used for immune cell analysis on day 9, 6 mice were performed for body weight, tumor growth and tissue histological analysis within 16 d, and remaining 8 mice were performed for survival curve study until day 100.

On day 9, the mice were sacrificed for immune activation study. The lymph nodes, tumor tissues, and spleens were acquired from 3 mice of every group and cut into as small pieces as possible. The pieces from each group were incubated with 3 mL PBS containing 5% fetal bovine serum (FBS), 3 mg collagenase type IV (Sigma-Aldrich), 0.3 mg hyaluronidase (Solarbio Science & Technology, Beijing), and 600 U deoxyribonuclease I (Solarbio Science & Technology, Beijing) for 2.5 h at 37 °C on a slow shaker. The obtained cell suspension was filtered with a cell strainer (70 µm), treated with RBC lysis reagent (Beyotime, China), and then washed with PBS containing 5% FBS for three times. The ratio of various immune cells was determined by flow cytometry analysis after labeled with corresponding antibodies. The FACS gating strategies are shown in Supplementary Figs. 55 and 56a.

The tumor volume and body weight of the mice were recorded throughout the treatment period. On day 16, all mice for tumor growing monitoring were harvested and the tumor tissues were collected, imaged, and fixed with paraformaldehyde (4%), followed by cell apoptosis and necrosis analyses via H&E, TUNEL (terminal deoxynucleotidyl transferase-mediated dUTP nick-end labeling), cPARP and cCASP3 assays. The various organs from mice were also dissected to make paraffin sections for further H&E staining. The survival of mice was monitored daily during the study. Once tumor reached ~1000 mm^3^, the mice were sacrificed  and the survival curves were obtained by counting the number of present mice. For the tumor rechallenge studies, treatment-naïve mice and cured mice from FKPN + irradiation treatment group were injected with 4T1 cells (1 × 10^6^ cells) in the opposite mammary fat pad. The tumor sizes of the mice were recorded every 5 days by caliper measurement.

#### Detection of abscopal effect

First, abscopal syngeneic models were built by injecting 1 × 10^6^ 4T1 cells into the right 4th mammary fat pad (primary tumor) and 0.5 × 10^6^ 4T1 cells into the left 4th mammary fat pad (distal tumor) of BALB/c mice. Once the primary tumor reached ~100 mm^3^ (day 0), the mice were allocated into 5 groups (*n* = 9). The mice were randomized based on sex, age, and weight. Different samples were subcutaneously injected into primary tumors and immediately treated with or without irradiation at an intensity of 0.3 W/cm^2^ (660 nm, 5 min) every 2 days four times. Then the distal tumor of three mice in each group was harvested for immune cell analyses by immunofluorescence as described above. The bilateral tumor sizes and body weight were recorded during the experiment. On day 15, the bilateral tumor tissues and organs were collected for H&E and TUNEL analyses.

#### DC and T-cell blocking

The BALB/c mice were randomized based on sex, age, and weight (five groups each containing three mice). For depletion of DCs, 200 μg anti-mouse CD11c antibodies or 200 μg mouse IgG (control) were intraperitoneally injected into mice on day −8 before the tumor inoculation, and the injections were repeated every 2 days until the day before the last treatment. For T-cell depletion, mice were intraperitoneally administered with anti-mouse CD4 (for CD4^+^ T-cell depletion, 200 μg/injection), anti-mouse CD8α (for CD8^+^ T-cell depletion, 200 μg/injection), or mouse IgG (control, 200 μg/injection) on day −2 before the first treatment, and once/6 d until the last treatment. The depletion efficiency of DCs, CD4^+^, and CD8^+^ T cell was measured by flow cytometry. The FACS gating strategies are shown in Supplementary Fig. 56b, c. The bilateral tumor sizes were monitored every 2 days during the experiment.

### Statistical and reproducibility

The results were reported as mean ± SD. The inter-group comparisons and data statistics were performed with Microsoft Excel, Origin 8.0 software, and GraphPad Prism 8. via student’s *t* test. **p < 0.01 and ***p < 0.001 were considered significant. Representative results showed in Figs. 2a, c–e, 3a, f, g, i, 4b, 6g, h, and Supplementary Figs. 1a, b, 4a, b, 13a, b, 15b, 17, 26a, b, 28, 30a–c, 35, 36, 38a, b, 39a, b, 46, 50 were generally obtained from three independent samples unless specified.

## Supplementary information

Supplementary Information

## Data Availability

Data supporting the findings of this work are available within the Article, Source Data and Supplementary Information files. Source data are provided in this paper.

## Source data

Source Data
