# Supplementary material for: A neutrophil mimicking metal-porphyrin-based nanodevice loaded with porcine pancreatic elastase for cancer therapy
Source: Nat Commun. 2023 Apr 8;14:1974. doi: 10.1038/s41467-023-37580-z (PMC10082843; doi:10.1038/s41467-023-37580-z)
Supplement: Supplementary file 1 — Supplementary Information [file 41467_2023_37580_MOESM1_ESM.pdf]

Supporting Information

A neutrophil mimicking metal-porphyrin-based nanodevice loaded with porcine pancreatic elastase for precise cancer therapy

Tingting Cui,[1,2] Yu Zhang,[1,2] Geng Qin,[1,2] Yue Wei,[1,2] Jie Yang,[1,2] Ying Huang, [1,2] Jinsong Ren,[1,2] and Xiaogang Qu,[1,2]*

AUTHOR ADDRESS

[1]State Key Laboratory of Rare Earth Resource Utilization and Laboratory of Chemical Biology, Changchun Institute of Applied Chemistry, Chinese Academy of Sciences, Changchun, Jilin 130022, P. R. China

[2]School of Applied Chemistry and Engineering, University of Science and Technology of China, Hefei, Anhui 230026, P. R. China

Supplementary 1. General Information

Reagents and materials

Meso-Tetra(4-carboxyphenyl) porphyrin (H_2TCPP, 97 % purity), ferric chloride ($FeCl_3 \cdot 6H_2O$, 99% purity), benzoic acid (BA, 99% purity), polyethylenimine (PEI, MW: 600 Da), and N,N-dimethylformamide (DMF, 99.9% purity) were purchased from Aladdin. The KRRRRG peptides were synthesized by Sangon Biotech (Shanghai) Co., Ltd. N-(3-Dimethylaminopropyl)-N′-ethylcarbodiimide hydrochloride (EDC, ≥98.0%), and N-hydroxysuccinimide (NHS, 98%), Trifluoroacetic acid (TFA,> 99%), dimethyl sulfoxide (DMSO), dichloromethane (DCM), porcine pancreatic elastase (PPE), N-methoxysuccinyl-Ala-Ala-Pro-Val p-nitroanilide (pNA), lipopolysaccharide (LPS), and 5,5'-dithiobis-(2-nitrobenzoic acid) (DTNB) were purchased from Sigma-Aldrich. Ethylene diamine tetraacetic acid (EDTA), Singlet Oxygen Sensor Green (SOSG), 2′,7′-Dichlorofluorescin diacetate (DCFH-DA), and fluorescein isothiocyanate (FITC) were purchased from Solarbio Science & Technology Co., Ltd. Mito-Tracker Red was supplied by Beyotime.

Supplementary Table

Antibody	Host species	Dilution	Duration	Supplier	Conjugation
β-actin	Mouse/Rabbit	1:1000	12 h	Bioss	N/A
β1-integrin	Rabbit	1:1000	12 h	Beyotime	N/A
L-selectin	Rabbit	1:1000	12 h	Beyotime	N/A
CXCR4	Rabbit	1:1000	12 h	Beyotime	N/A
Fas/CD95	Rabbit	1:500/1:1000	4+12 h/12 h	Proteintech	N/A
Fas/CD95	Rabbit	1:500/1:1000	4+12 h/12 h	Biorbyt	N/A
Histone H1.0	Rabbit	1:200	4+12 h/12 h	Beyotime	N/A
H2AX	Rabbit	1:1000	12 h	Biorbyt	N/A
H2AX	Rabbit	1:1000	12 h	Beyotime	N/A
γH2AX	Rabbit	1:1000	12 h	Beyotime	N/A
CD11c	Armenian Hamster	1:16	30 min	Lianke Bio	PE
CD80	Armenian Hamster	1:40	30 min	Lianke Bio	FITC
CD86	Rabbit	1:32	30 min	Lianke Bio	APC
CD45	Rabbit	1:16	30 min	Biolegend	PE
CD8	Rabbit	1:16	30 min	Biolegend	APC
CD3	Rabbit	1:50	30 min	Biolegend	FITC
CD4	Rabbit	1:16	30 min	Biolegend	APC
PARP	Mouse	1:1000	12 h	Biolegend	N/A
cPARP	Rabbit	1:1000	12 h	Beyotime	N/A
PARP	Rabbit	1:1000	12 h	Xinyu Biology	N/A
cPARP	Rabbit	1:1000	12 h	Xinyu Biology	N/A
CASP3	Rabbit	1:1000	12 h	Beyotime	N/A
cCASP3	Rabbit	1:1000	12 h	Beyotime	N/A
CD8	Rabbit	1:200	4+12 h	Leinco	N/A
CD4	Rabbit	1:200	4+12 h	Beyotime	N/A
Anti Mouse IgG (H+L)	Goat	1:500	30 min	Beyotime	Cy3/AF488
Anti Rabbit IgG (H+L)	Goat	1:500	30 min	Beyotime	Cy3/AF488
CD8α	Rabbit	1:1		Tonbo	N/A
CD4	Rabbit	1:1		Tonbo	N/A
CD11c	Armenian Hamster	1:1		Leinco	N/A
CD44	Rabbit	1:200	30 min	Tonbo	PE
CD62L	Rabbit	1:200	30 min	Tonbo	FITC
CD44	Rabbit	1:200	30 min	Tonbo	FITC
ICAM-1	Mouse	1:20	1 h	Tonbo	FITC
ICAM-1	Rabbit	1:20	1 h	Leinco	FITC
CD44	Rabbit	1:1	2 h	Tonbo	N/A
ICAM-1	Mouse	1:1	2 h	Tonbo	N/A
ICAM-1	Rabbit	1:1	2 h	Leinco	N/A
Goat Anti-Mouse/Rabbit IgG (H+L)		1:1000	2 h	Fine Biotech	HRP

Table 1. Antibodies used for this work.

Supplementary 2. Supplementary Figures

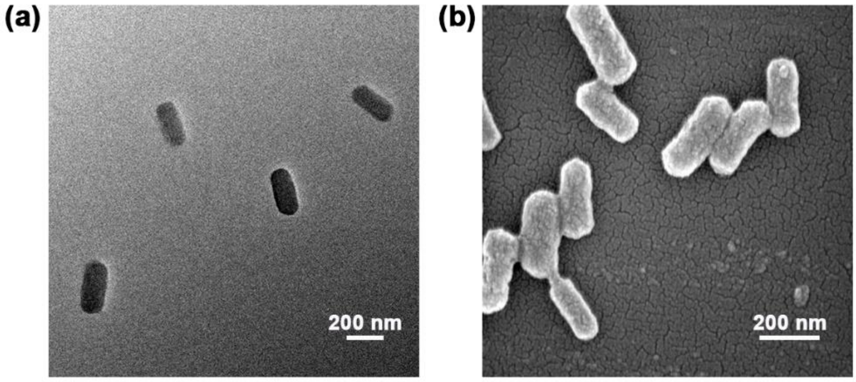

Supplementary Figure 1. The morphology of Fe-porphyrin MOF. Scanning electron microscopy (SEM) image (a) and transmission electron microscopy (TEM) image (b) of Fe-porphyrin MOF. (a, b) Representative of n = 3 independent experiments.

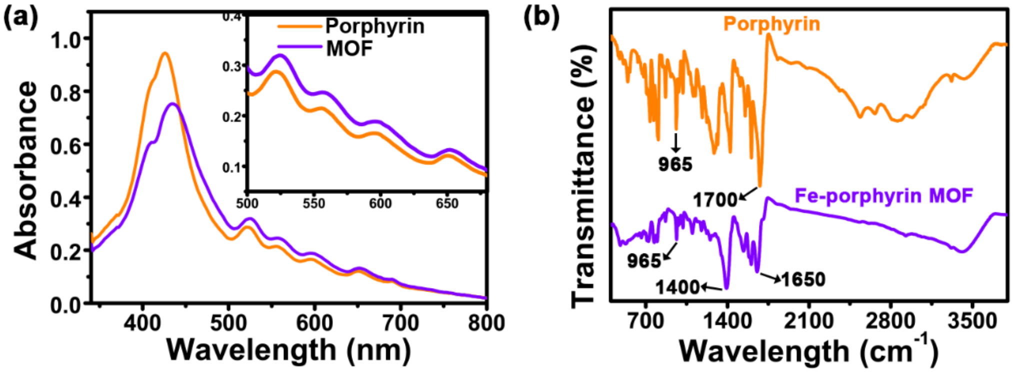

Supplementary Figure 2. The characterization of synthesized Fe-porphyrin MOF. (a) UV−vis absorption spectra of porphyrin and Fe-porphyrin MOF. (b) Fourier transforms infrared (FTIR) spectra of porphyrin and Fe-porphyrin MOF. Source data are provided as a Source Data file.

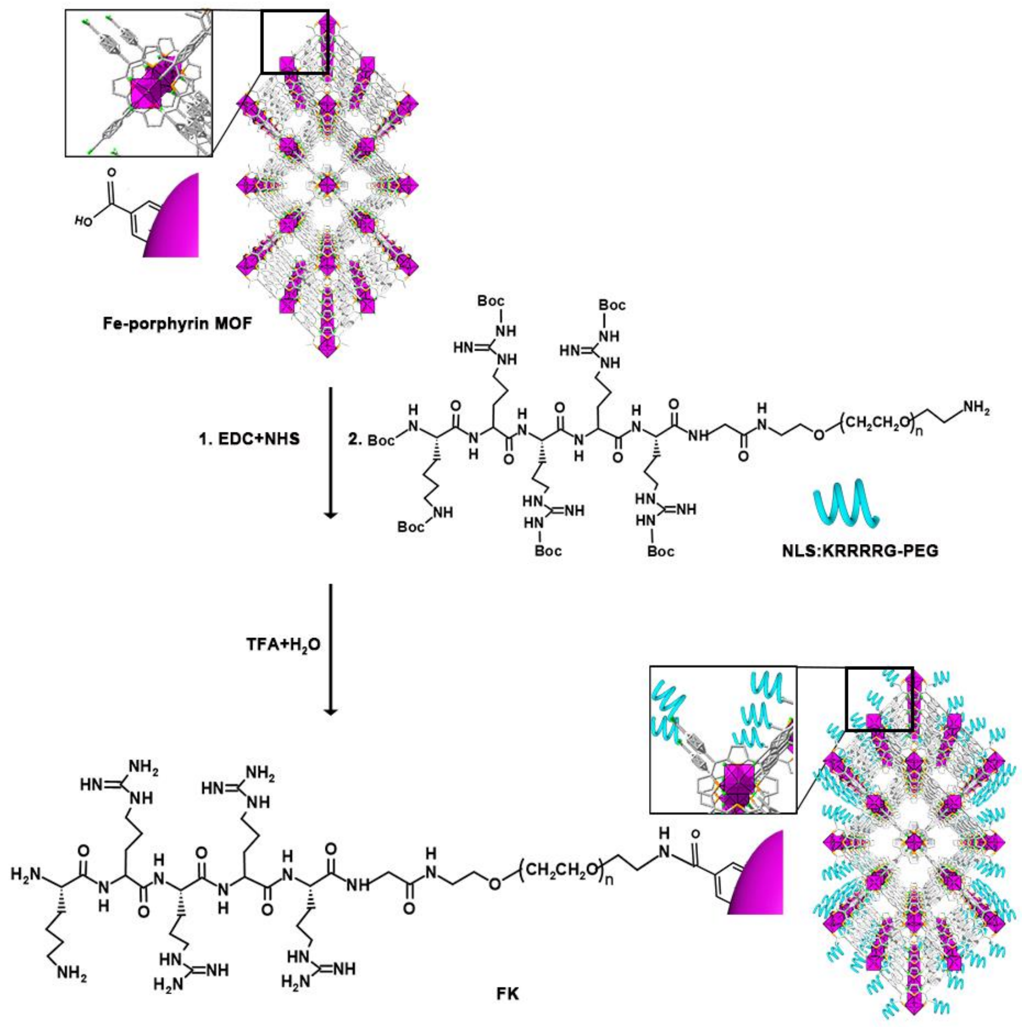

Supplementary Figure 3. Conjugation of Fe-porphyrin MOF with peptide.

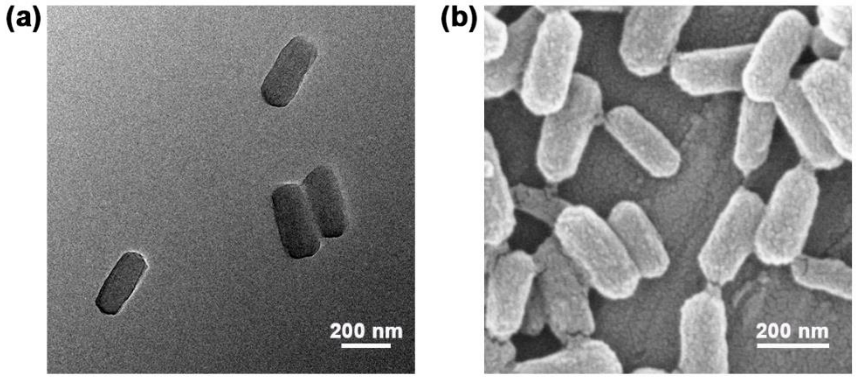

Supplementary Figure 4. The morphology characterization of FK. SEM image (a) and TME image (b) of FK. (a, b) Representative of n = 3 independent experiments.

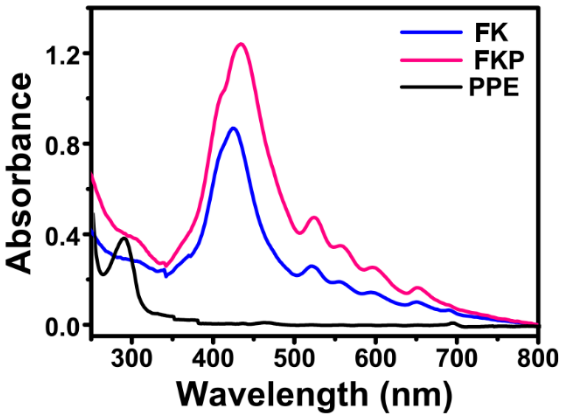

Supplementary Figure 5. UV−vis absorption spectra of prepared particles and PPE.

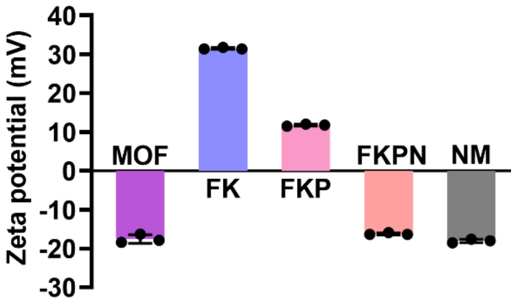

Supplementary Figure 6. Zeta-potential of synthesized particles. Data were presented as mean ± SD (n = 3 independent experiments). Source data are provided as a Source Data file.

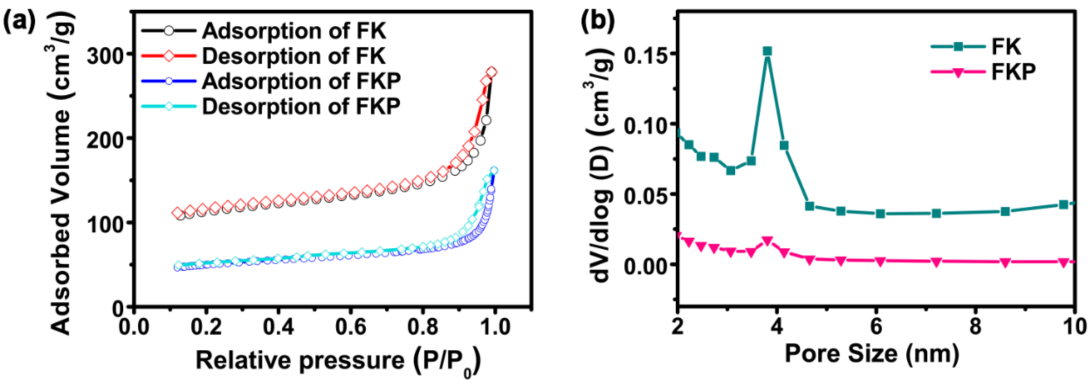

Supplementary Figure 7. The protein loading of FKP assessed by nitrogen adsorption experiments. Nitrogen adsorption and desorption isotherms (a) and pore size distribution (b) of FK and FKP. Source data are provided as a Source Data file.

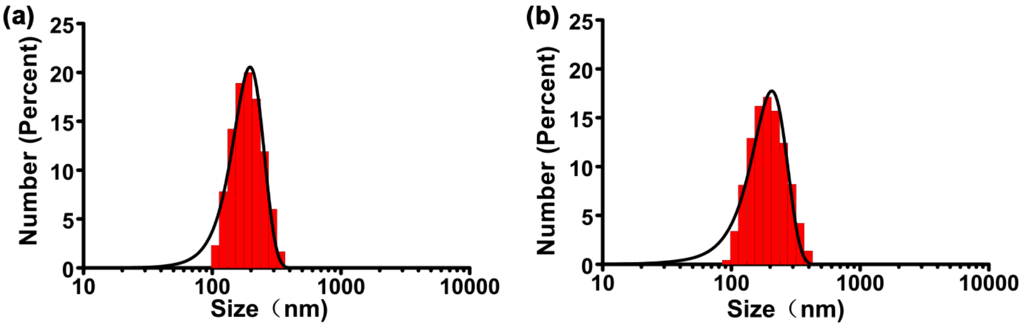

Supplementary Figure 8. The size distribution of different particles. Size distribution histogram of the (a) Fe-porphyrin MOF and (b) FK. Source data are provided as a Source Data file.

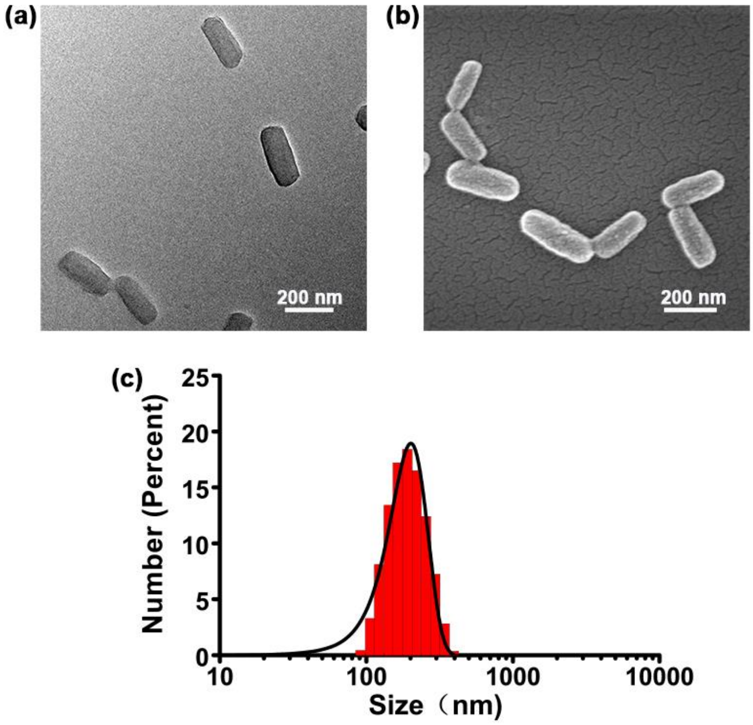

Supplementary Figure 9. The morphology and size distribution of FKP. SEM image (a) TEM image (b) and size distribution histogram (c) of the FKP. Source data are provided as a Source Data file.

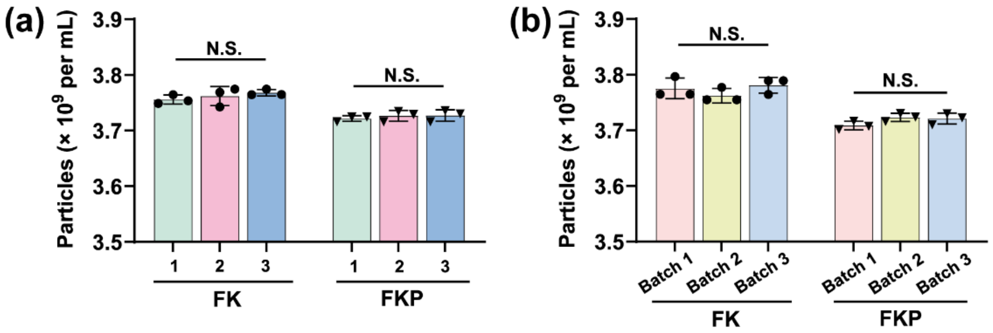

Supplementary Figure 10. The FK and FKP particle concentrations determined by flow cytometer analysis. (a) Flow cytometric analysis of FK and FKP particles within one prepared batch. The samples in groups 1, 2 and 3 were taken from the same batch of particles. Data were presented as mean ± SD (n = 3 independent measurements using the same sample). N.S. indicates non-significant statistical differences. (b) Flow cytometric analysis of FK and FKP particles from three independent prepared batches. Data were presented as mean ± SD (n = 3 independent measurements using the same sample). Significance was assessed using Student's t test (two-tailed). N.S. indicates non-significant statistical differences. Source data are provided as a Source Data file.

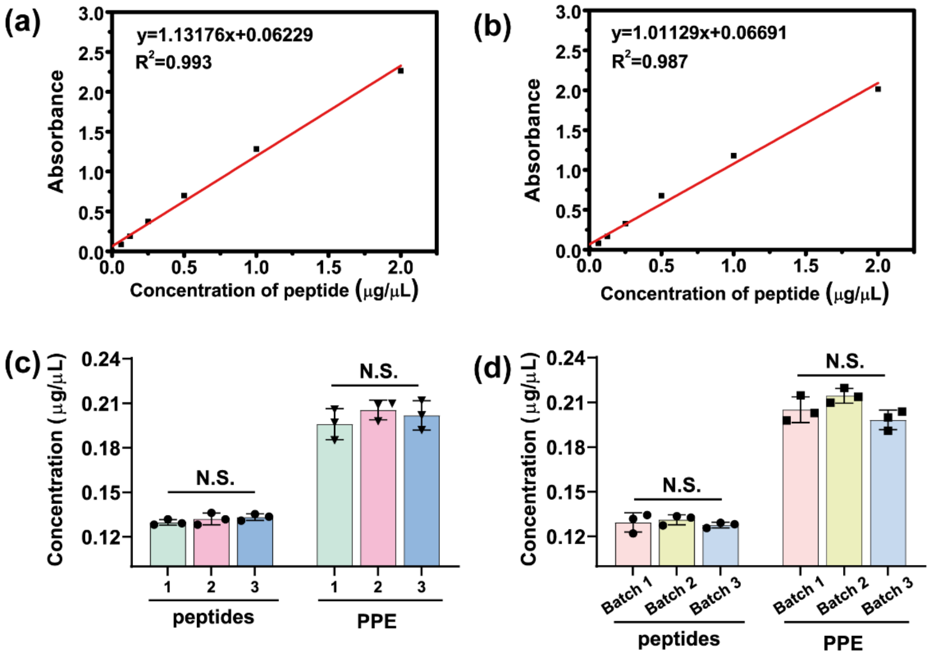

Supplementary Figure 11. The loading efficiency of peptide or PPE on the FK and FKP particles. Standard curve of (a) peptide and (b) PPE. (c) BCA protein assay analysis of peptide or PPE concentrations in as-collected supernatants from one prepared batchone batch. The samples in groups 1, 2 and 3 were taken from the same batch of supernatants. Data were presented as mean ± SD (n = 3 independent measurements using the same sample). N.S. indicates non-significant statistical differences. (d) BCA protein assay analysis of peptide or PPE concentrations in as-collected supernatants from three independent prepared batches. Data were presented as mean ± SD (n = 3 independent measurements using the same sample). Significance was assessed using Student's t test (two-tailed). N.S. indicates non-significant statistical differences. Source data are provided as a Source Data file.

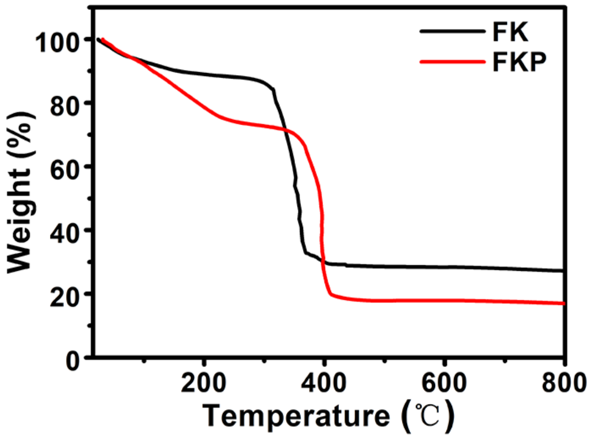

Supplementary Figure 12. Thermo gravimetric analysis (TGA) for PPE loading efficiency. TGA of synthesized particles. Source data are provided as a Source Data file.

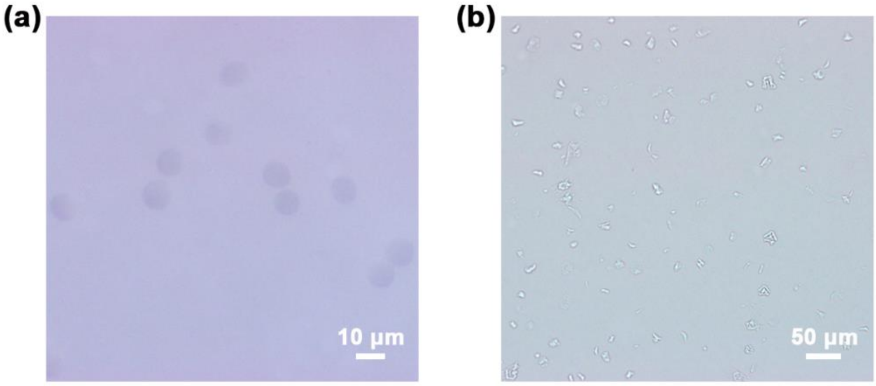

Supplementary Figure 13. The purified Neutrophils and NM fragments. (a) The image of intact neutrophils. (b) The image of cell membrane debris. They were observed by optical microscopy. (a, b) Representative of n = 3 independent experiments.

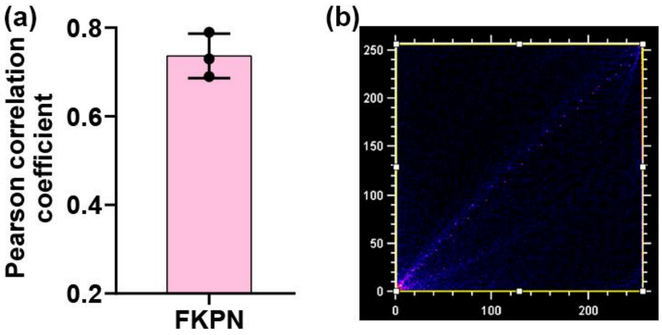

Supplementary Figure 14. The successful PPE loading and membrane coating of FKPN. (a) Pearson correlation coefficient and (b) representative scatter plot of colocalization of PPE and membrane coating in Figure 1c. Data were presented as mean ± SD (n = 3 independent experiments). Source data are provided as a Source Data file.

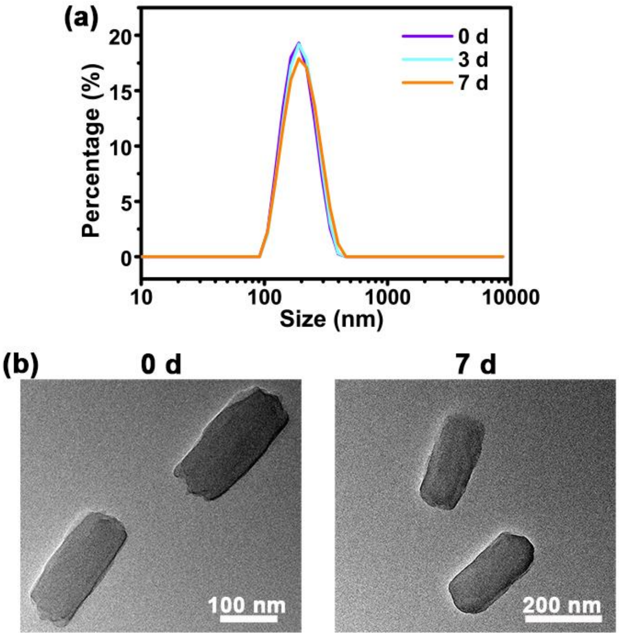

Supplementary Figure 15. The stability study of FKPN. Dynamic light scattering (DLS) analysis (a) and TEM images (b) of FKPN after incubation with PBS for various times. Source data are provided as a Source Data file. (a, b) Representative of n = 3 independent experiments.

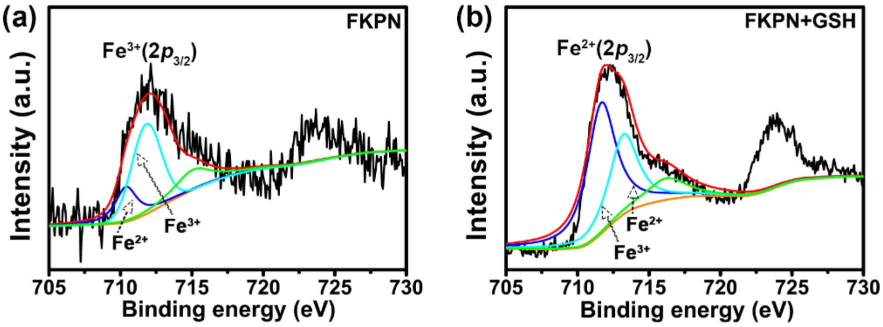

Supplementary Figure 16. GSH-triggered FKPN unlocking assessed by XPS. XPS high-resolution spectra of Fe 2*p* in FKPN before and after cultivation with GSH. Source data are provided as a Source Data file.

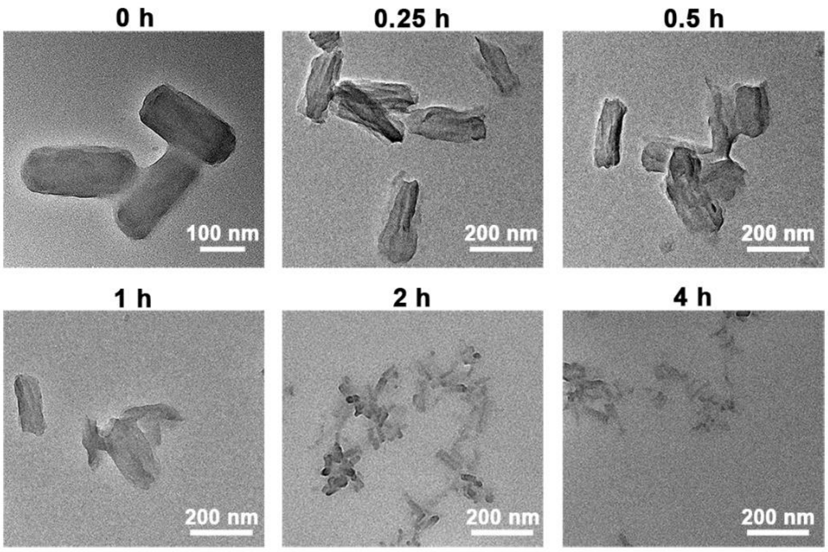

Supplementary Figure 17. GSH-responsive FKPN degradation assessed by TEM. TEM images of the FKPN nanodevice after incubated with GSH with different time interval. (a, b) Representative of n = 3 independent experiments.

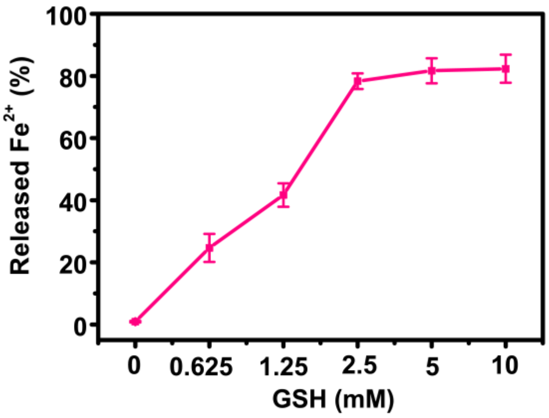

Supplementary Figure 18. The percentage of Fe released from FKPN incubated with different concentrations of GSH. Data were presented as mean ± SD (n = 3 independent experiments). Source data are provided as a Source Data file.

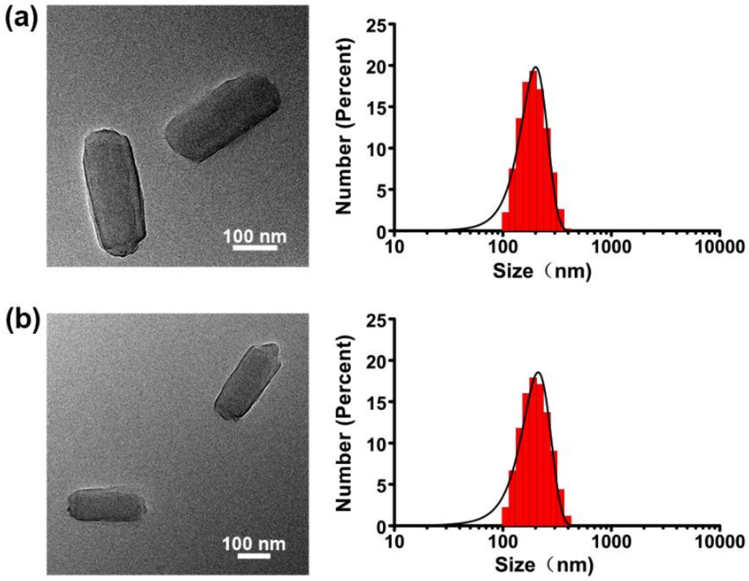

Supplementary Figure 19. The morphology and size distribution of FKN and FPN. TEM images and size distribution histogram of (a) FKN and (b) FPN. (a, b) Representative of n = 3 independent experiments. Source data are provided as a Source Data file.

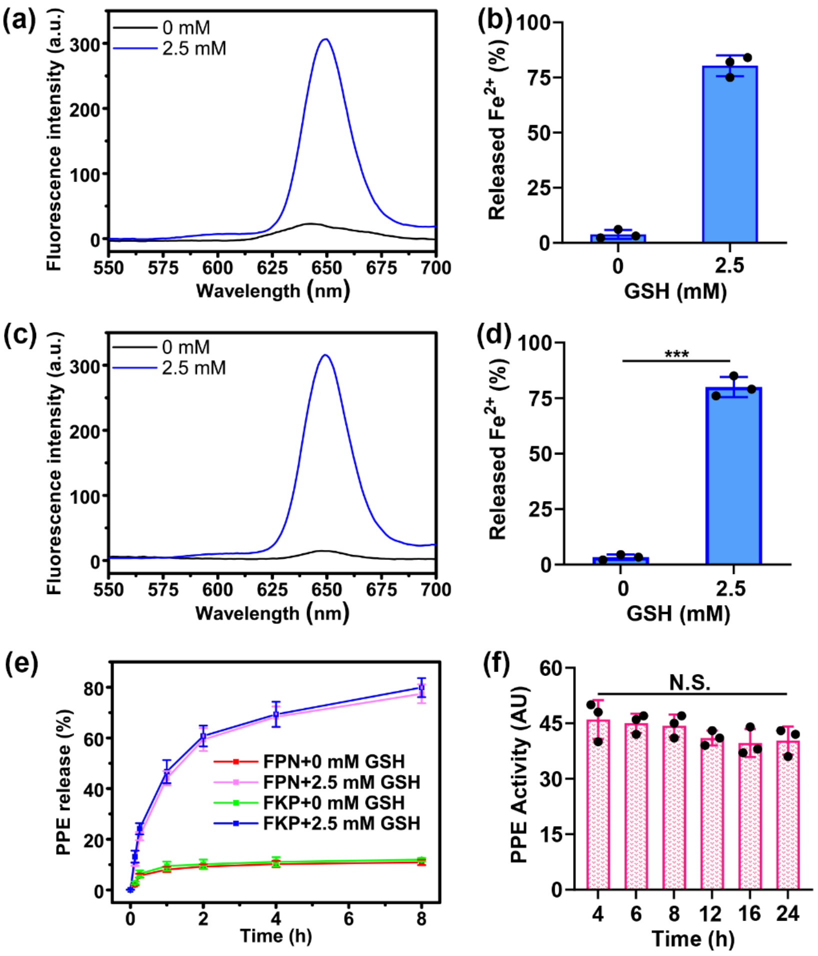

Supplementary Figure 20. The characterizations of FKP and FPN. Fluorescence changes of (a) FKN and (c) FPN with GSH (2.5 mM) treatments. The percentage of Fe released from (b) FKN or (d) FPN incubated with GSH. (e) The release profile of PPE from FPN and FKP treated by GSH for various times. Data were presented as mean ± SD (n = 3 independent experiments). Significance was assessed using Student's t test (two-tailed). P values: 9.63787E-06 for the percentage of Fe released from FPN incubated with 0 mM GSH vs 2.5 mM GSH. (***$p < 0.001$). N.S. indicates non-significant statistical differences. Source data are provided as a Source Data file.

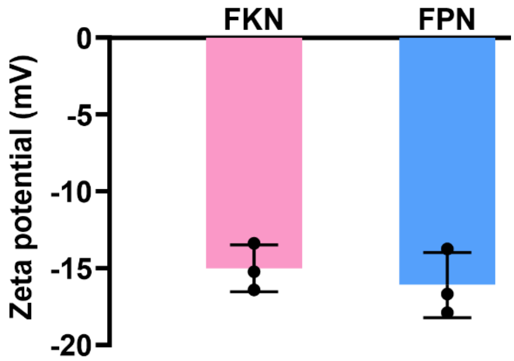

Supplementary Figure 21. Zeta-potential of FKN and FPN. Data were presented as mean ± SD (n = 3 independent experiments). Source data are provided as a Source Data file.

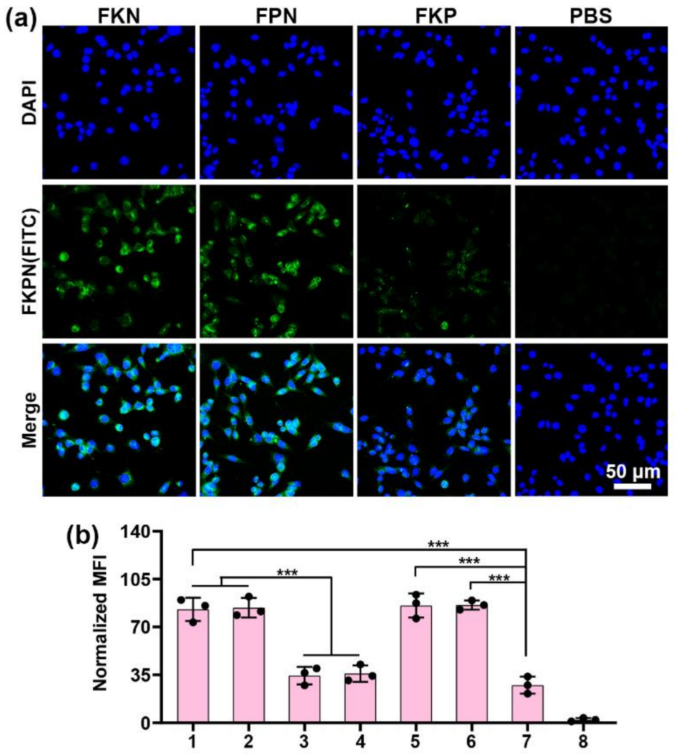

Supplementary Figure 22. The cellular uptake of different nanoparticles. (a) Fluorescence images of MDA-MB-231 cells after treatment with different formulations for 4 h. Representative of n = 3 independent experiments. (b) Calculations of normalized mean fluorescence intensity (MFI) in cancer and non-cancer cells treated with different samples for 4 h. 1: MDA-MB-231/FKPN; 2: 4T1/FKPN; 3: MCF-10A/FKPN; 4: HC11/FKPN; 5: MDA-MB-231/FKN; 6: MDA-MB-231/FPN; 7: MDA-MB-231/FKP; 8: MDA-MB-231/PBS. Data were presented as mean ± SD (n = 3 independent experiments. Significance was assessed using Student's t test (two-tailed). P values: 0.000133342 for MDA-MB-231/FKPN and 4T1/FKPN groups vs MCF-10A/FKPN and HC11/FKPN groups; 0.000815023 for MDA-MB-231/FKPN group vs MDA-MB-231/FKP group; 0.000735865 for MDA-MB-231/FKN group vs MDA-MB-231/FKP group; 0.000139049 for MDA-MB-231/FPN group vs MDA-MB-231/FKP group. (***$p < 0.001$). Source data are provided as a Source Data file.

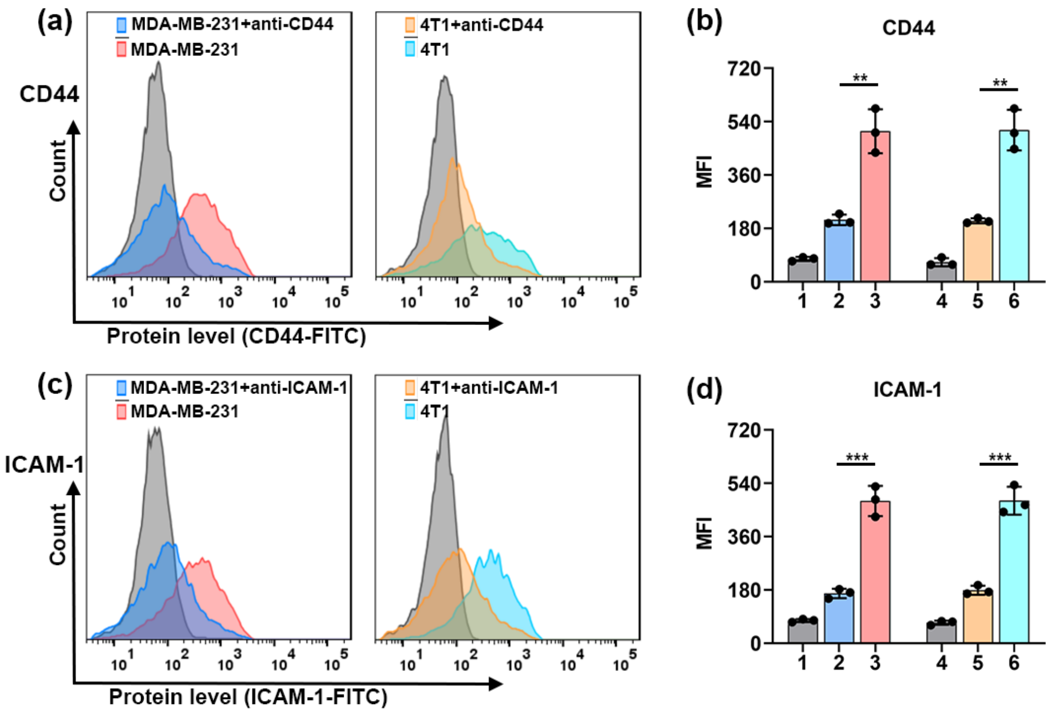

Supplementary Figure 23. The antibody blocking assay for evaluation of effect of membrane proteins on FKPN. Flow cytometry analysis and calculations of adhesion proteins (a, b) CD44 and (c, d) ICAM-1 on MDA-MB-231 and 4T1 cells. The cells were preincubated with anti-CD44 and anti-ICAM-1 antibodies to block these adhesion proteins for antibody blocking assay. Cells without incubation with any antibodies were taken as control (grey). 1: blank MDA-MB-231 cells; 2: MDA-MB-231 cells pretreated with anti-CD44 or anti-ICAM-1 antibodies; 3: MDA-MB-231 cells; 4: blank 4T1 cells; 5: 4T1 cells pretreated with anti-CD44 or anti-ICAM-1 antibodies; 6: 4T1 cells. Data were presented as mean ± SD (n = 3 independent experiments). Significance was assessed using Student's t test (two-tailed). P values: 0.00244668 for MDA-MB-231 cells pretreated with anti-CD44 vs MDA-MB-231 cells; 0.00154976 for 4T1 cells pretreated with anti-CD44 vs 4T1 cells; 0.000539093 for MDA-MB-231 cells pretreated with anti-ICAM-1 vs MDA-MB-231 cells; 0.000452878 for 4T1 cells pretreated with anti-ICAM-1 vs 4T1 cells. (**$p < 0.01$, ***$p < 0.001$). Source data are provided as a Source Data file.

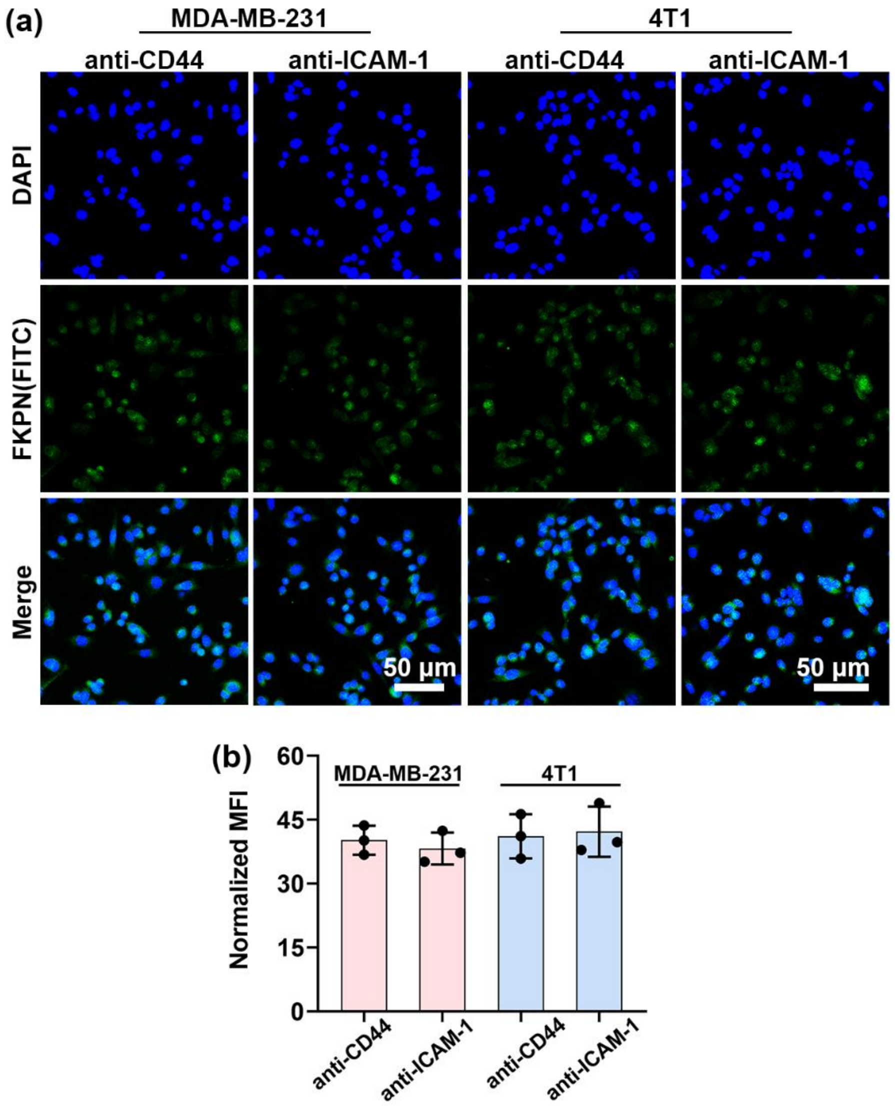

Supplementary Figure 24. The cellular uptake of FKPN after antibody blocking. (a) Fluorescence images of MDA-MB-231 and 4T1 cells treated with FKPN for 4 h following antibody blocking. Representative of n = 3 independent experiments. (b) Calculations of normalized MFI in treated MDA-MB-231 and 4T1 cells. Data were presented as mean ± SD (n = 3 independent experiments). Source data are provided as a Source Data file.

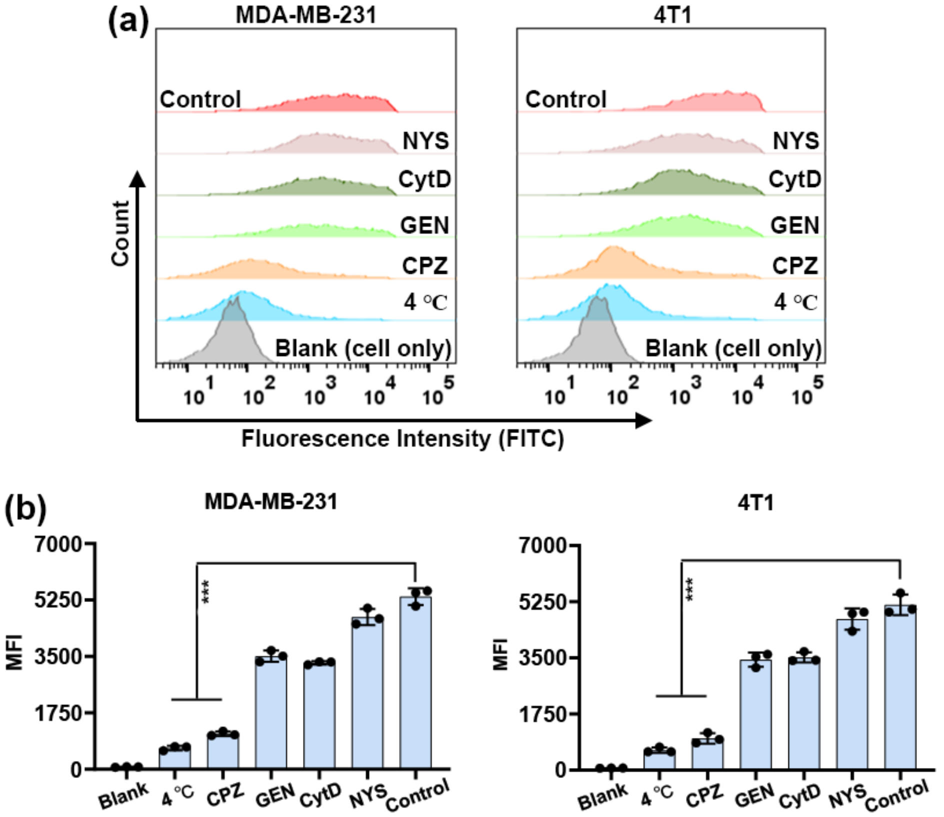

Supplementary Figure 25. The internalization pathway studies of FKPN. (a) Flow cytometry analysis and (b) fluorescence quantification of cellular uptake of FKPN in MDA-MB-231 and 4T1 cells treated with low temperature (4 °C), chlorpromazine (CPZ), genistein (GEN), cytochalasin D (CytD) and nystatin (NYS). Data were presented as mean ± SD (n = 3 independent experiments). Significance was assessed using Student's t test (two-tailed). P values: 2.68831E-05 for MDA-MB-231 treated with low temperature (4 °C) and chlorpromazine (CPZ) vs control group; 0.000108582 for 4T1 cells treated with low temperature (4 °C) and chlorpromazine (CPZ) vs control group. (***$p < 0.001$). Source data are provided as a Source Data file.

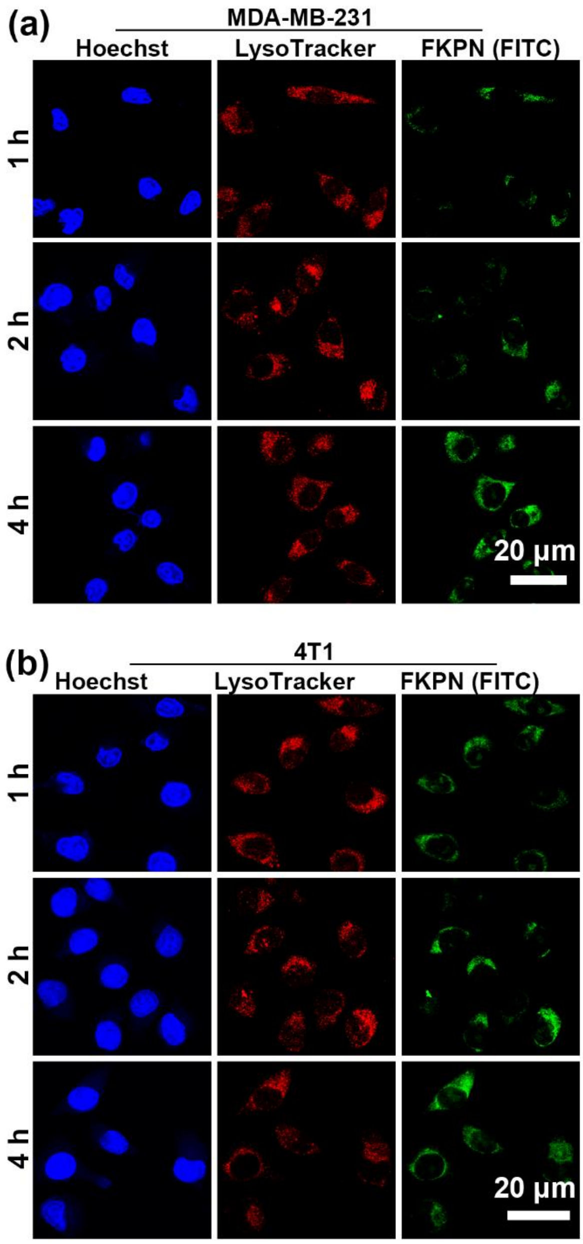

Supplementary Figure 26. The effective escape of FKPN from lysosomes. Different fluorescent channels of (a) MDA-MB-231 and (b) 4T1 cells in Figure 2b. The nuclei were stained using Hoechst 33342 (blue) and lysosomes were stained with LysoTracker red. (a, b) Representative of n = 3 independent experiments.

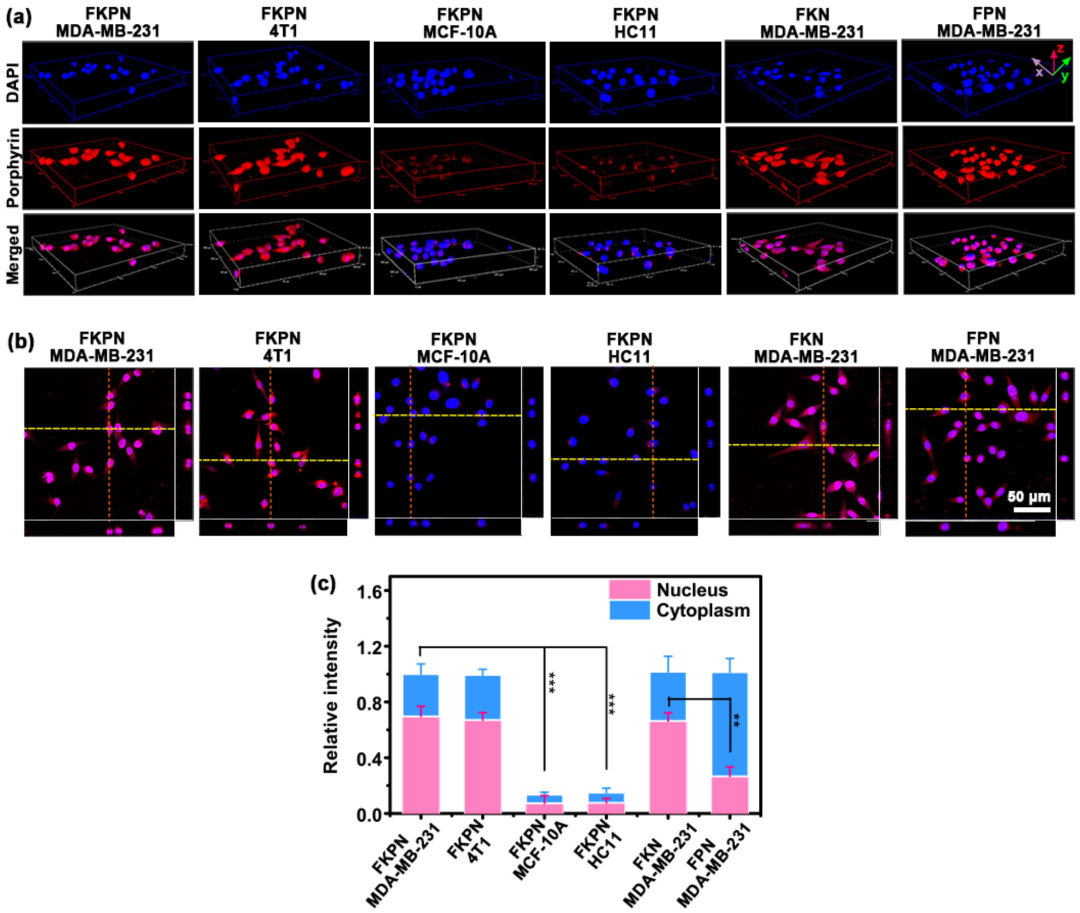

Supplementary Figure 27. The cancer cell-selective uptake and disintegration of particles. (a) 3D-CLSM images of cancer and non-cancer cells after co-incubation with different particles for 4 h. (b) The corresponding CLSM photomicrograph with YZ (cells on the orange line) and XZ (cells on the yellow line) planes of cancer and non-cancer after co-incubation with different particles. (c) Calculations of nuclear and cytoplasmic porphyrin fluorescence intensity in treated cells. Data were presented as mean ± SD (n = 3 independent experiments). Significance was assessed using Student's t test (two-tailed). P values: 6.39289E-06 for MDA-MB-231/FKPN group vs MCF-10A/FKPN group; 1.14401E-11 for MDA-MB-231/FKPN group vs and HC11/FKPN group; 0.001496281 for nuclear porphyrin fluorescence intensity in MDA-MB-231/FKN group vs MDA-MB-231/FPN group. (**$p < 0.01$, ***$p < 0.001$). (a, b) Representative of n = 3 independent experiments. Source data are provided as a Source Data file.

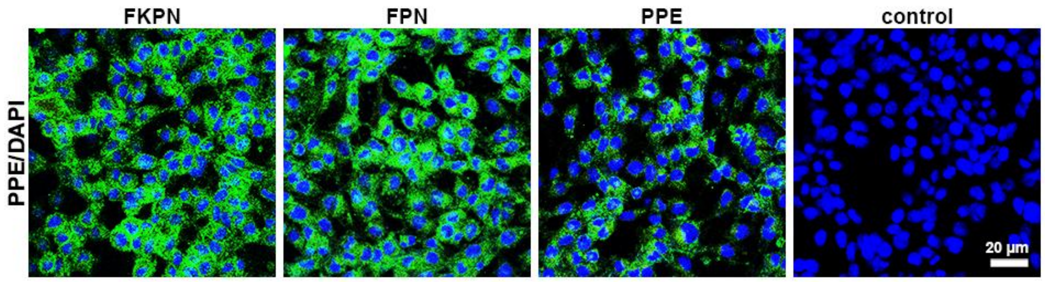

Supplementary Figure 28. The enhanced PPE internalization induced by nanoparticles with membrane camouflage. Fluorescence images of MDA-MB-231 cells incubated with FKPN, FPN, free PPE and PBS for 4 h. The nuclei were stained using DAPI (blue) and PPE was labeled with FITC (green). Representative of n = 3 independent experiments.

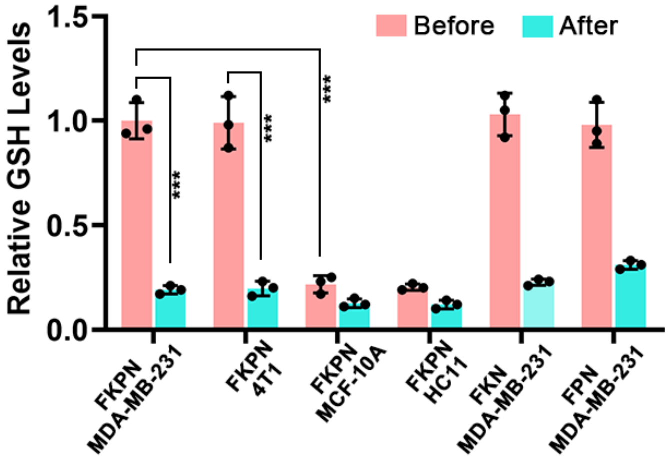

Supplementary Figure 29. Detection of intracellular GSH levels in cancer and non-cancer cells after treatment with different samples. Data were presented as mean ± SD (n = 3 independent experiments). Significance was assessed using Student's t test (two-tailed). P values: 9.64876E-05 for intracellular GSH levels in MDA-MB-231 cells before vs after treatment with FKPN; 0.000455031 intracellular GSH levels in 4T1 cells before vs after treatment with FKPN; 0.000149162 for intracellular GSH levels in MDA-MB-231 cells vs 4T1 cells before treatment with FKPN. (***$p < 0.001$). Source data are provided as a Source Data file.

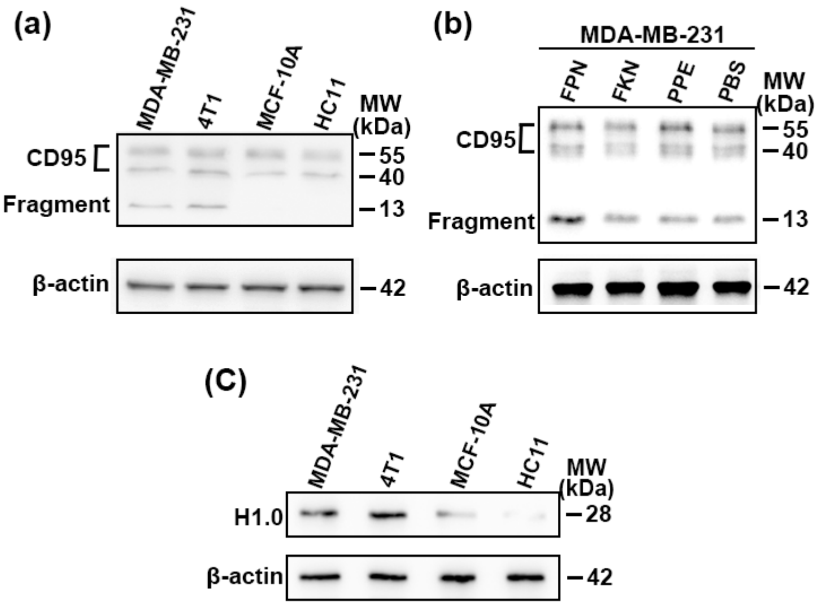

Supplementary Figure 30. The liberation of CD95 DD and the elevated histone H1 isoforms in cancer cells. (a) Effect of FKPN on CD95 cleavage in cancer and non-cancer cells assessed by western blot analysis. Blot is representative of n = 2 independent experiments. (b) Effect of different samples on CD95 cleavage in MDA-MB-231 cells assessed by western blot analysis. Blot is representative of n = 2 independent experiments. (c) Western blot analysis of histone H1.0 isoforms in cancer and non-cancer cells. Blot is representative of n = 2 independent experiments. Source data are provided as a Source Data file.

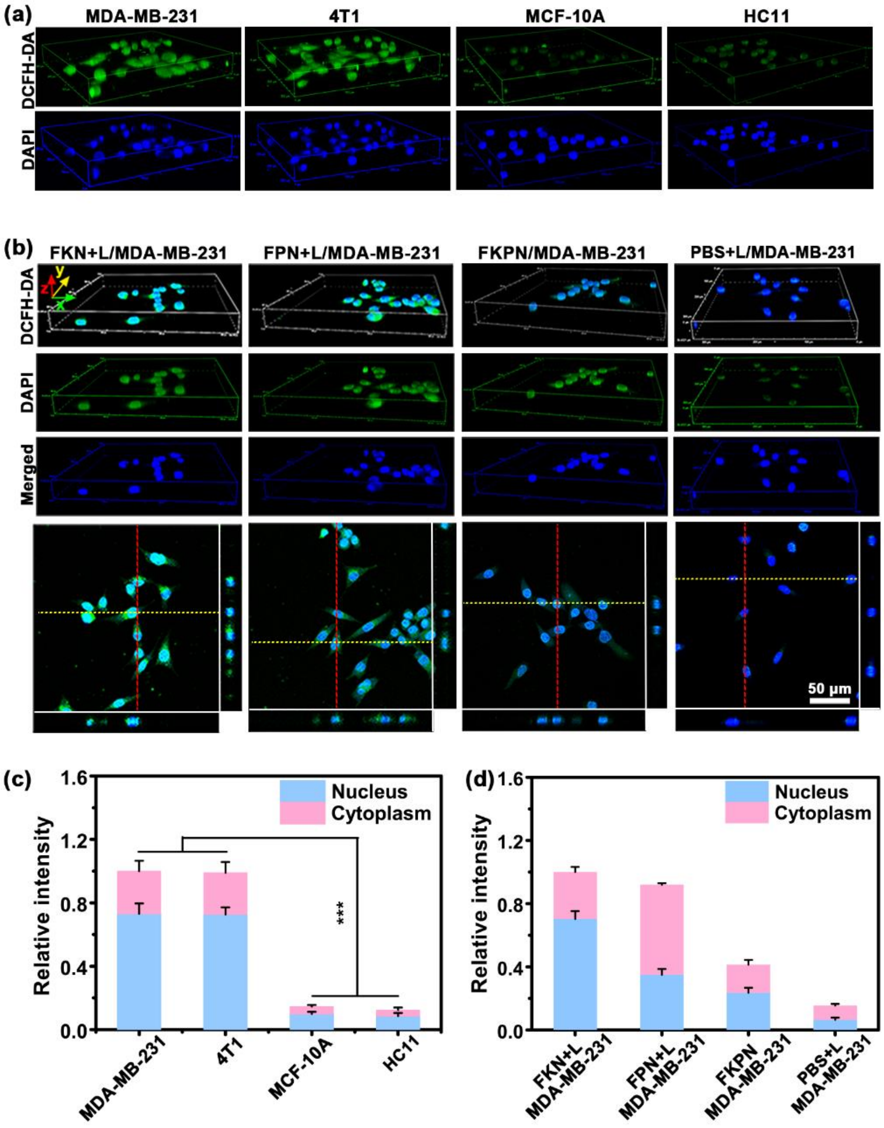

Supplementary Figure 31. The nuclear $^1O_2$ generation assessed by 3D-CLSM. (a) 3D-CLSM images of cancer and non-cancer cells after co-incubation with FKPN and treated with laser irradiation. (b) 3D-CLSM images of MDA-MB-231 cells after treatment with different samples and treated with or without laser irradiation. The corresponding CLSM photomicrograph with YZ (cells on the red line) and XZ (cells on the yellow line) planes of treated MDA-MB-231 cells. (c) Calculations of nuclear and cytoplasmic fluorescence intensity in treated cells in (a). Significance was assessed using Student's t test (two-tailed). P values: 4.20551E-07 for calculations of nuclear and cytoplasmic fluorescence intensity in MDA-MB-231 and 4T1 cells vs in MCF-10A and HC11 cells. (***$p < 0.001$). (d) Calculations of nuclear and cytoplasmic fluorescence intensity in treated cells in (b). Data were presented as mean ± SD (n = 3 independent experiments). (a, b) Representative of n = 3 independent experiments. Source data are provided as a Source Data file.

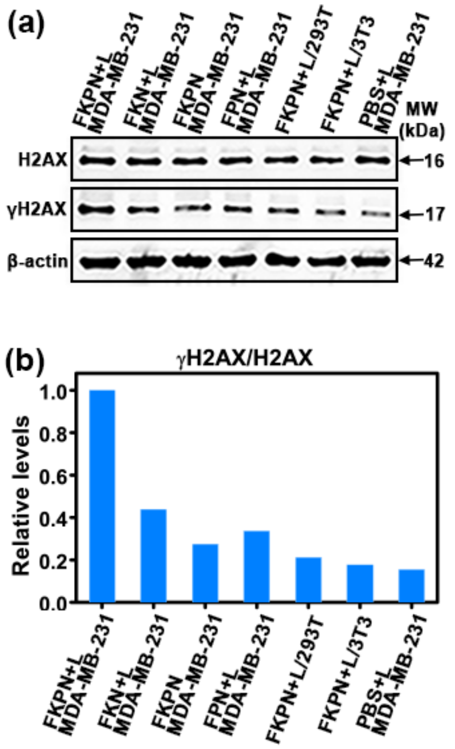

Supplementary Figure 32. The DNA DSBs evaluation by western blotting. (a) Western blot analysis of γH2AX and H2AX protein in cancer and normal cells after treatment with different formulations. Blot is representative of n = 2 independent experiments. (b) Calculations of the γH2AX/H2AX ratio in cancer and non-cancer cells. Source data are provided as a Source Data file.

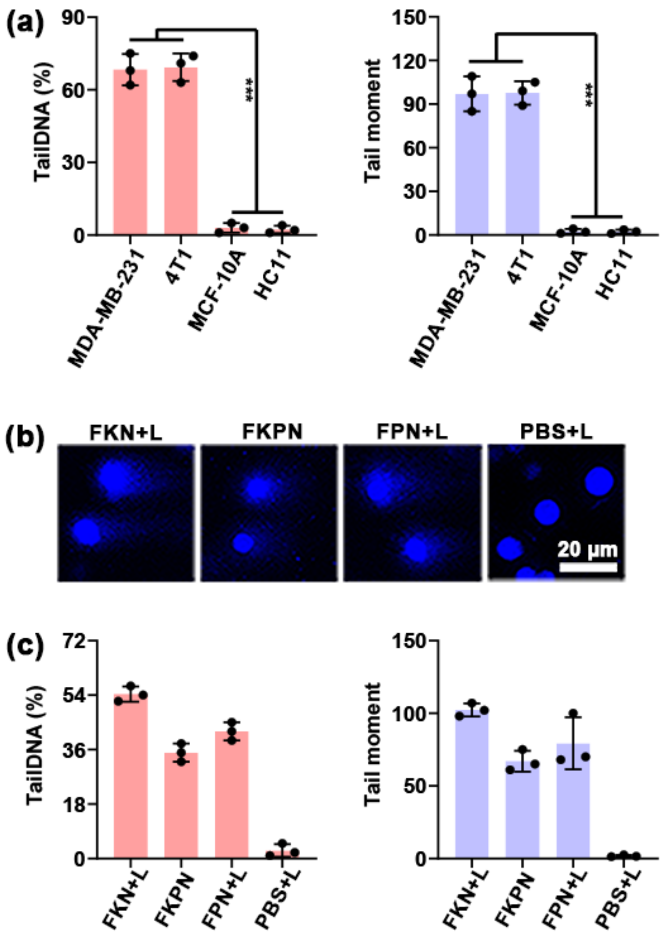

Supplementary Figure 33. The DNA damage assessment using the comet assays. (a) Calculations of comet tailDNA and tail moment of cancer and non-cancer cells in Figure 2g. Data were presented as mean ± SD (n = 3 independent experiments). Significance was assessed using Student's t test (two-tailed). P values: 1.07591E-05 for calculations of comet tailDNA in MDA-MB-231 and 4T1 cells vs in MCF-10A and HC11 cells; 4.2055E-05 for calculations of tail moment in MDA-MB-231 and 4T1 cells vs in MCF-10A and HC11 cells. (***$p < 0.001$). (b) Fluorescent images of DNA damages of MDA-MB-231 cells in different groups using the comet assays. Representative of n = 3 independent experiments. (c) Calculations of comet tailDNA and tail moment of MDA-MB-231 cells in (b). Data were presented as mean ± SD (n = 3 independent experiments). Source data are provided as a Source Data file.

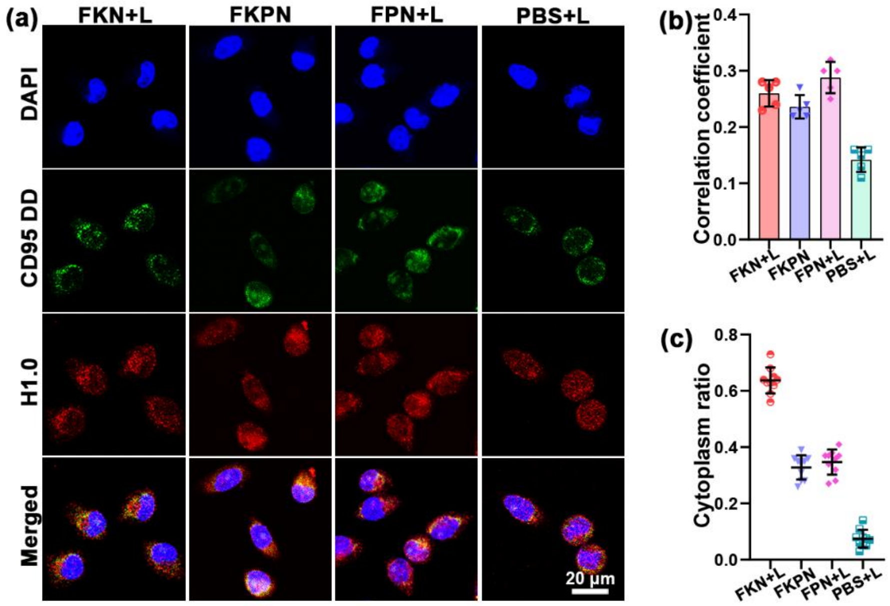

Supplementary Figure 34. The cytoplasmic histone H1.0 localization induced by different treatments. (a) Immunofluorescence images of MDA-MB-231 cells stained for CD95 DD, H1.0 and nuclei. Representative of n = 3 independent experiments. (b) Calculations of correlation coefficient between CD95 DD and H1.0 in (a). (c) Corresponding calculations of cytoplasmic H1.0 ratio in treated cells in (a). Data were presented as mean ± SD (n = 10 or 5 independent experiments). Source data are provided as a Source Data file.

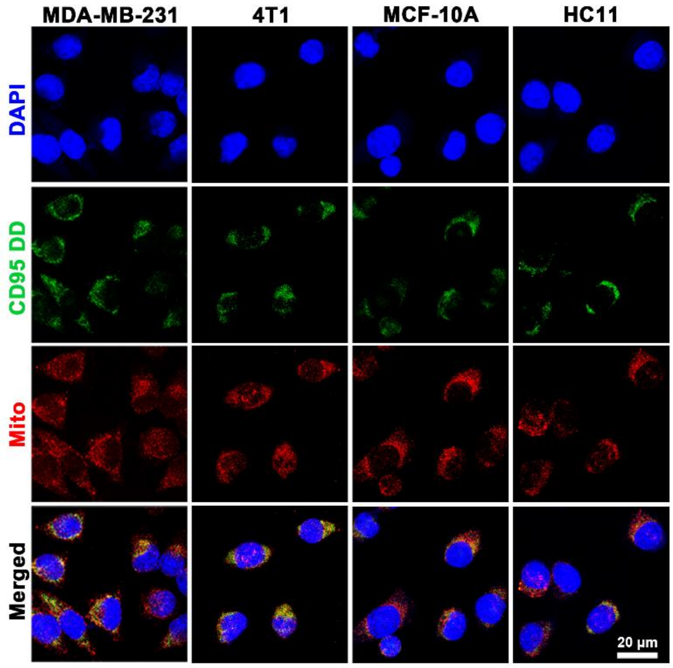

Supplementary Figure 35. The co-localization of CD95 DD-H1.0 with mitochondria. Immunofluorescence staining of CD95 DD, mitochondria and nuclei in cancer and non-cancer cells after treatment with FKPN and laser irradiation. Representative of n = 3 independent experiments.

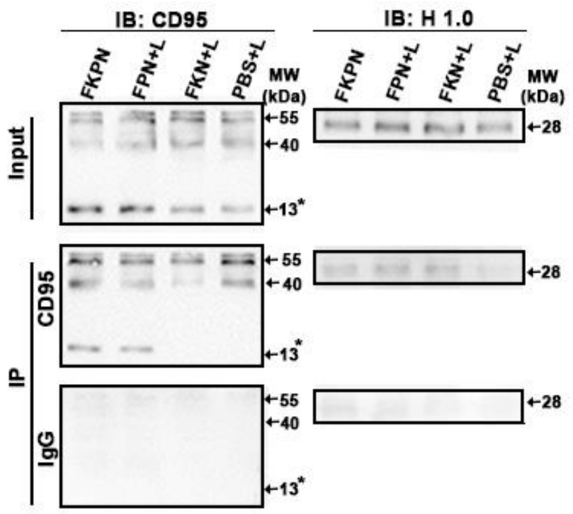

Supplementary Figure 36. Co-immunoprecipitation analysis of CD95 DD with H1.0 in MDA-MB-231 cells treated with different formulations. Blot is representative of n = 2 independent experiments. Source data are provided as a Source Data file.

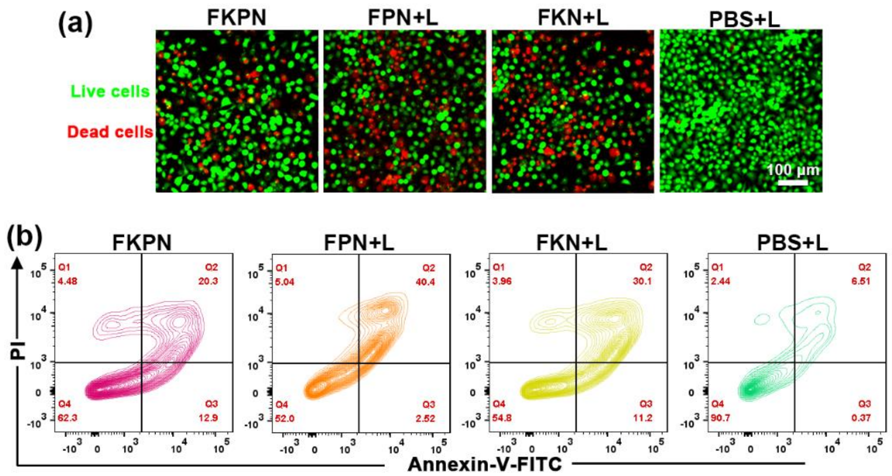

Supplementary Figure 37. The targeted killing effect of FKPN plus laser irradiation. (a) CLSM images of live/dead staining of treated MDA-MB-231 cells in different groups. Green: calcein AM-labeled live cells; red: propidium iodide (PI)-labeled dead cells. (b) Flow cytometry analysis of the apoptosis of MDA-MB-231 cells staining with Annexin V-FITC/PI after treatment with different formulations. (a, b) Representative of n = 3 independent experiments.

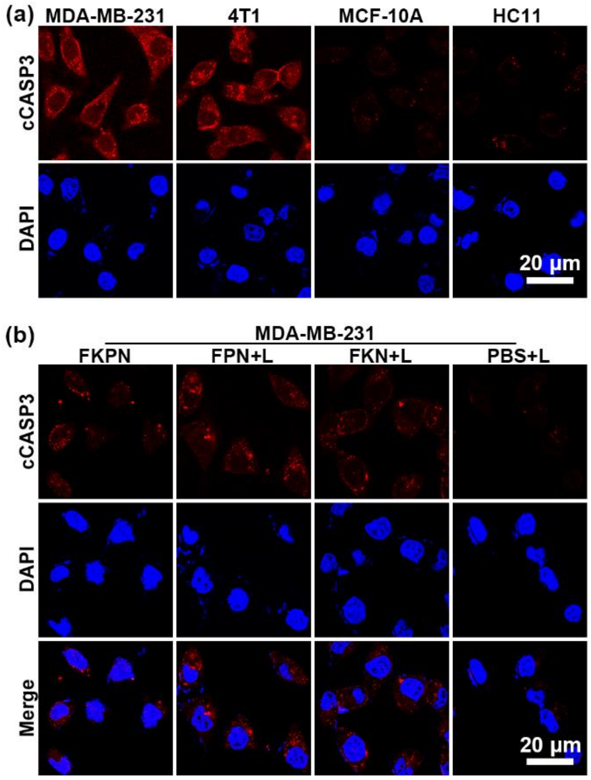

Supplementary Figure 38. The activation of apoptosis effector CASP3 in cancer cells. (a) Different fluorescent channels of cancer and non-cancer cells stained for cCASP3 (red) and nuclei (blue) in Figure 4d. (b) Apoptosis pathway analysis of treated MDA-MB-231 cells assessed by immunofluorescence staining of cCASP3. (a, b) Representative of n = 3 independent experiments.

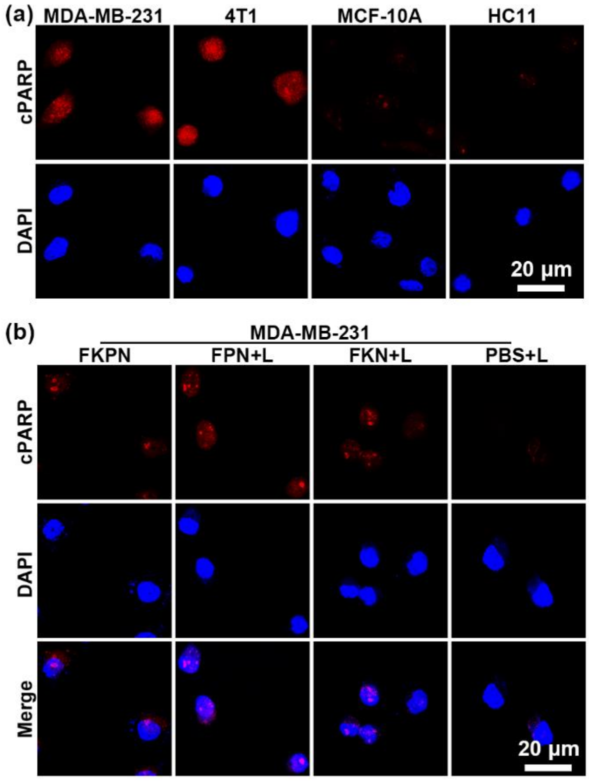

Supplementary Figure 39. The activation of apoptosis effector PARP in cancer cells. (a) Different fluorescent channels of cancer and non-cancer cells stained for cPARP (red) and nuclei (blue) in Figure 4d. (b) Apoptosis pathway analysis of treated MDA-MB-231 cells assessed by immunofluorescence staining of cPARP. (a, b) Representative of n = 3 independent experiments.

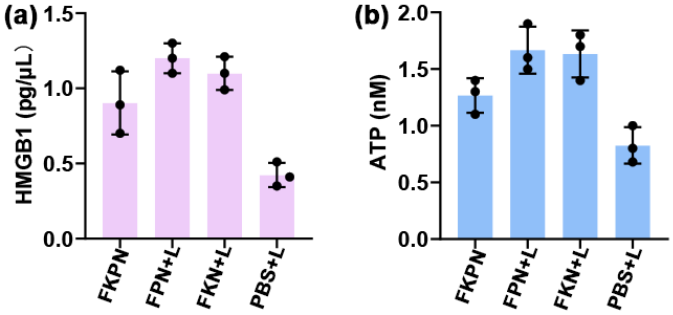

Supplementary Figure 40. The induction of ICD effect by different treatments. (a) Quantification of released HMGB1 from MDA-MB-231 cells in all groups. (b) Amount of released ATP from MDA-MB-231 cells after different treatments. Data were presented as mean ± SD (n = 3 independent experiments). Source data are provided as a Source Data file.

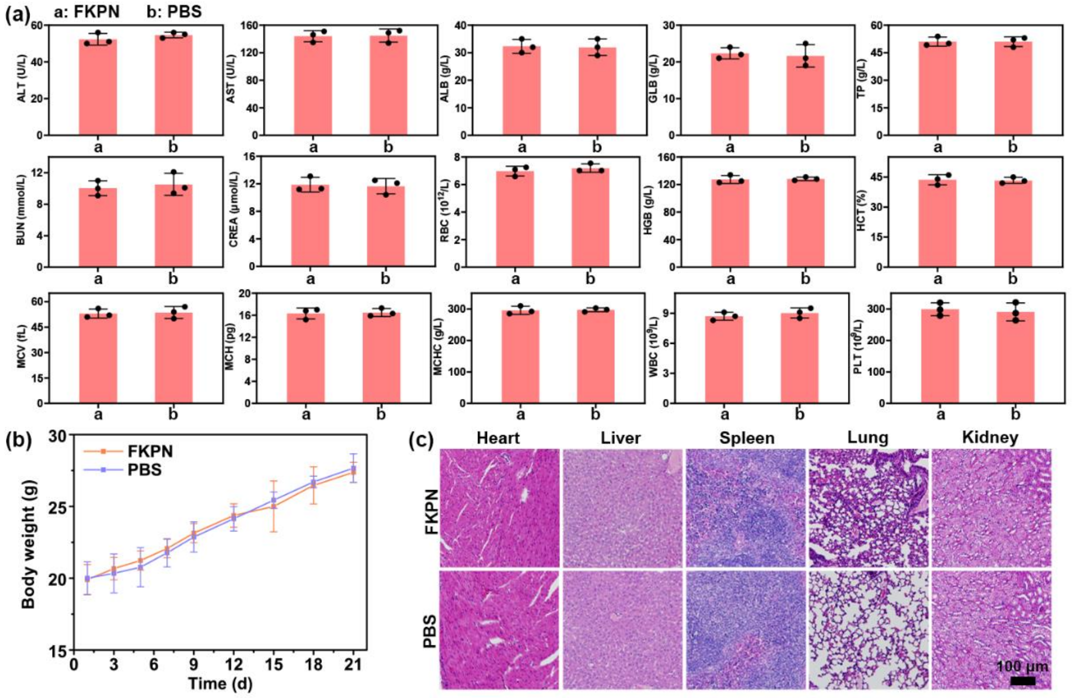

Supplementary Figure 41. The biosecurity of FKPN. (a) Blood serum biochemical and hematological analysis of the healthy mice intravenously injected with FKPN and PBS. Data were presented as mean ± SD (n = 3 mice). (b) Body weight changes of mice treated with FKPN and PBS. Error bars represent standard deviations (n = 3 mice). (c) Micrographs of H&E stained main organs (including liver, spleen, lung, kidney) of treated mice. Source data are provided as a Source Data file.

FKPN: $A_t=352.68e^{(-t/4.61)}+150.36$
$R^2=0.96331$ $t_{1/2}=4.98$ h

FPN: $A_t=361.88e^{(-t/3.74)}+149.62$
$R^2=0.96754$ $t_{1/2}=4.06$ h

FKN: $A_t=367.03e^{(-t/4.43)}+139.36$
$R^2=0.96068$ $t_{1/2}=4.51$ h

FKP: $A_t=439.43e^{(-t/1.65)}+84.84$
$R^2=0.95963$ $t_{1/2}=1.43$ h

Supplementary Figure 42. *In vivo* pharmacokinetic studies. *In vivo* pharmacokinetic parameters for NPs in 4T1 tumour-bearing mice.

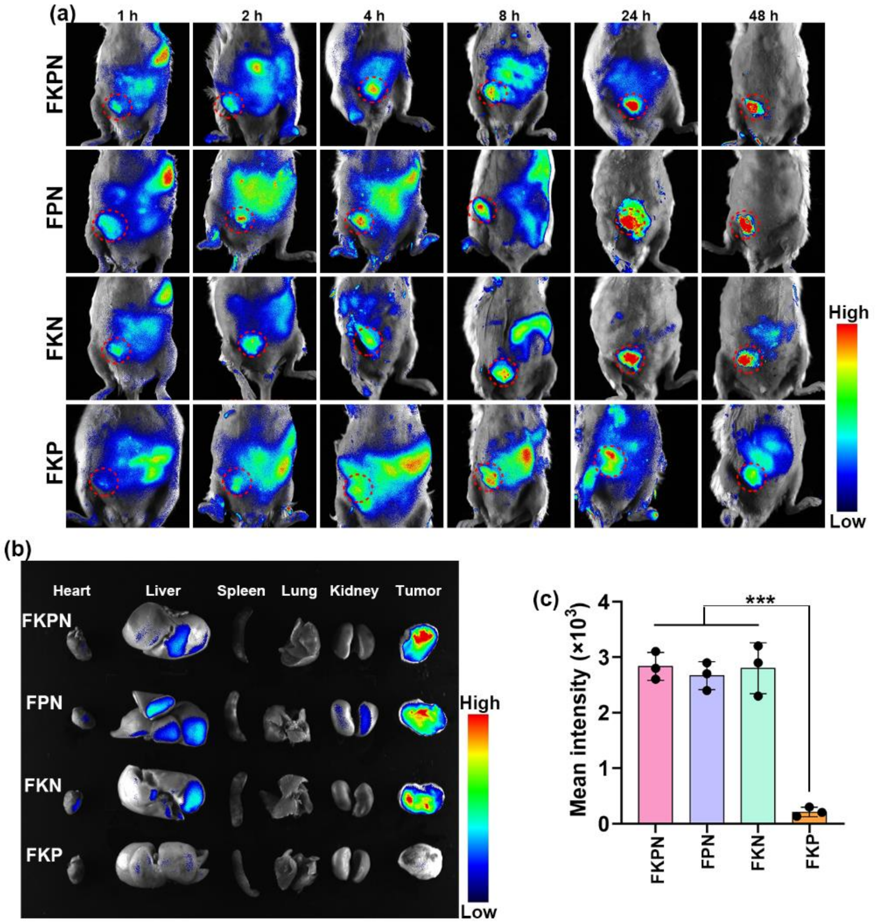

Supplementary Figure 43. The tumor targeting and accumulation capability of FKPN. (a) *In vivo* fluorescence images of tumor-bearing mice after injected with different particles for different times. (b) Ex vivo fluorescent imaging of tumors and major organs from mice at 24 h post-injection. (c) Quantification of the fluorescence signal of tumor tissues in (b). Data were presented as mean ± SD (n = 3 mice). Significance was assessed using Student's t test (two-tailed). P values: 2.92034E-06 for quantification of the fluorescence signal of tumor tissues in FKPN, FPN and FKN groups vs in FKP group. (***$p < 0.001$). Source data are provided as a Source Data file.

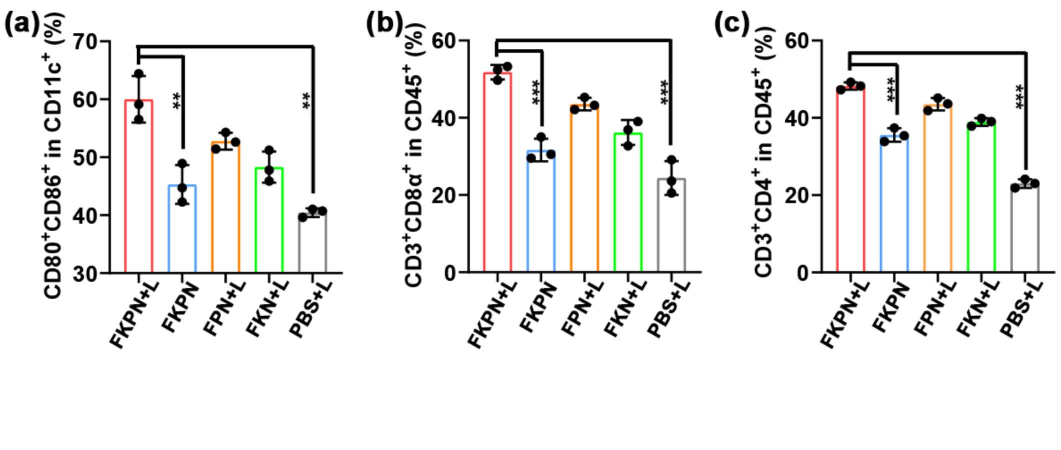

Supplementary Figure 44. Quantitative analysis of activated immune cells. (a) Proportion of matured DCs in the lymph nodes. Proportion of tumor-infiltrating (b) $CD8^+$ T cells and (c) $CD4^+$ T cells. Data were presented as mean ± SD (n = 3 mice). Significance was assessed using Student's t test (two-tailed). P values: 0.007280797 for matured DCs in FKPN+L group vs in FKPN group; 0.001161314 for matured DCs in FKPN+L group vs in PBS+L group; 0.000556007 for $CD8^+$ T cells in FKPN+L group vs in FKPN group; 0.000559938 for $CD8^+$ T cells in FKPN+L group vs in PBS+L group; 0.000389827 for $CD4^+$ T cells in in FKPN+L group vs in FKPN group; 7.29037E-06 for CD4+ T cells in FKPN+L group vs in PBS+L group. (**p < 0.01, ***p < 0.001). Source data are provided as a Source Data file.

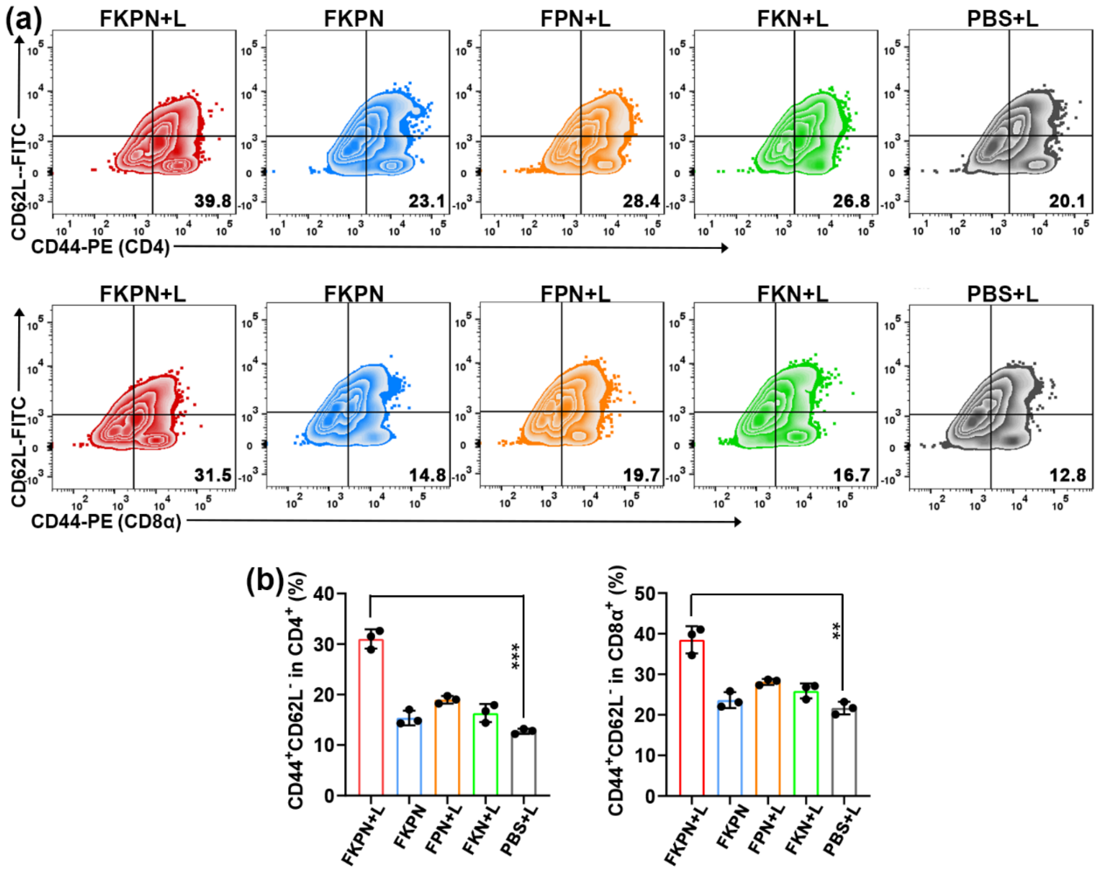

Supplementary Figure 45. The activation of effector memory T cells. (a) Flow cytometry plots of effector memory T cells ($CD44^+CD62L^-CD4^+$, $CD44^+CD62L^-CD8^+$) derived from spleens after various treatments. (b) The proportions of $CD44^+CD62L^-$ cells in the total $CD4^+$ T cells or CD8+ T cells. Data were presented as mean ± SD (n = 3 mice). Significance was assessed using Student's t test (two-tailed). P values: 8.71171E-05 for $CD44^+CD62L^-CD4^+$ T cells in FKPN+L group vs in PBS+L group; 0.001383391 for $CD44^+CD62L^-CD8^+$ T cells in FKPN+L group vs in PBS+L group. (**p < 0.01, ***p < 0.001). Source data are provided as a Source Data file.

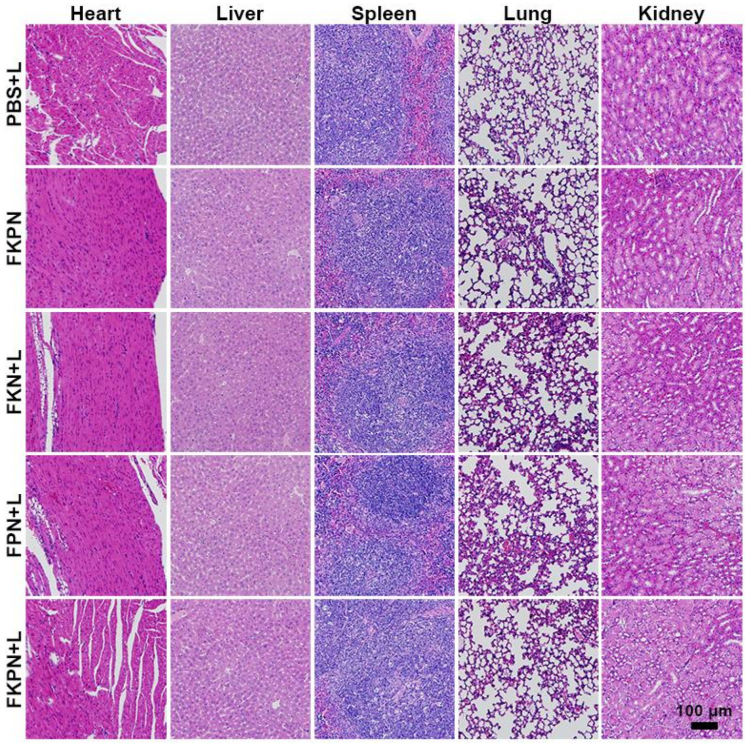

Supplementary Figure 46. Biosafety assessment of different treatments. Micrographs of H&E stained main organs (including Heart, liver, spleen, lung, kidney) of each group of mice after 16 days of different treatments. Representative of n = 3 independent experiments.

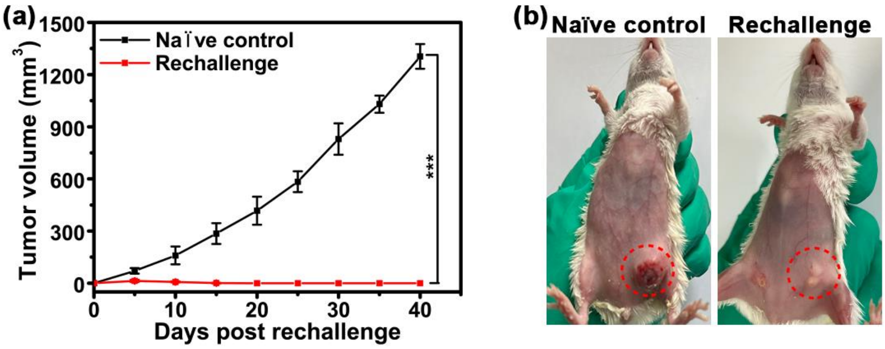

Supplementary Figure 47. The immune memory effect induced by FKPN plus laser irradiation. (a) Tumor volume in treatment-naïve or previously cured mice rechallenged by subcutaneous injection of 4T1 cells (1×10^6). (b) A representative image of treatment-naïve and rechallenged mouse after 40 days of tumor inoculation. Data were presented as mean ± SD (n = 4 mice). Significance was assessed using Student's t test (two-tailed). P values: 2.78644E-08 for tumor volume in treatment-naïve group vs rechallenge group. (***$p < 0.001$). Source data are provided as a Source Data file.

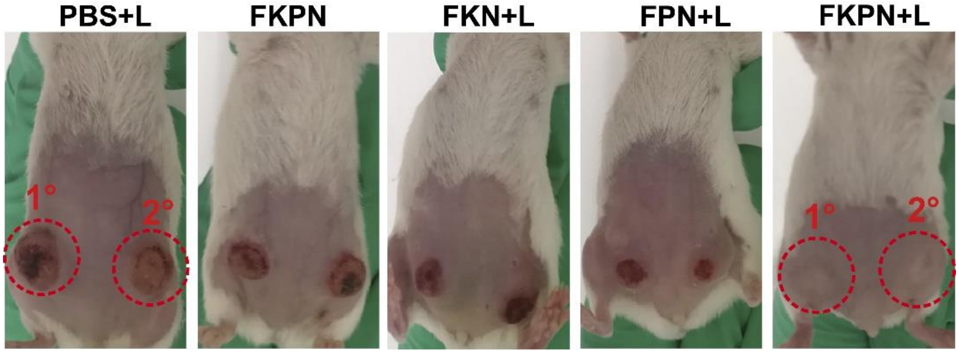

Supplementary Figure 48. The abscopal effect of different treatment formulations. The representative photographs on day 14 of bilateral 4T1 tumor-bearing mice with different treatments.

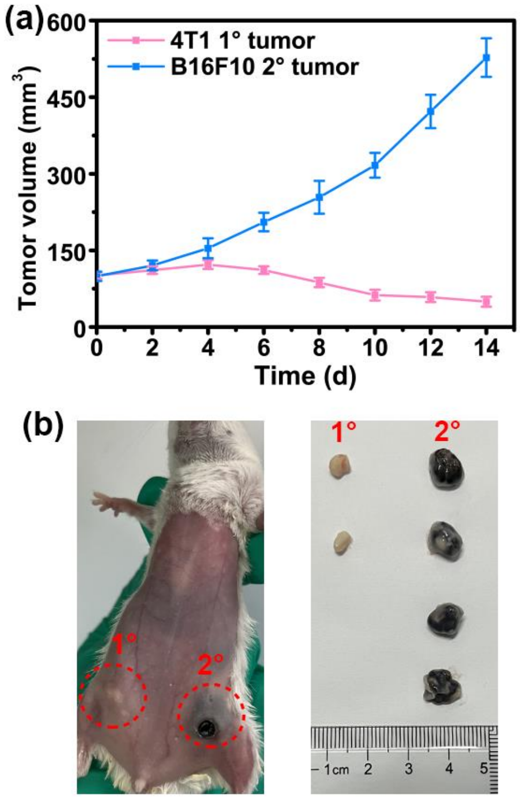

Supplementary Figure 49. The tumor specificity of abscopal effect. (a) Primary and distant tumors growth curves of mice with genetically distinct primary and distant tumors. Data were presented as mean ± SD (n = 4 mice). (b) Representative photographs of mouse and the tumors excised from tumor-bearing mice on day 14. (n = 4 mice). Source data are provided as a Source Data file.

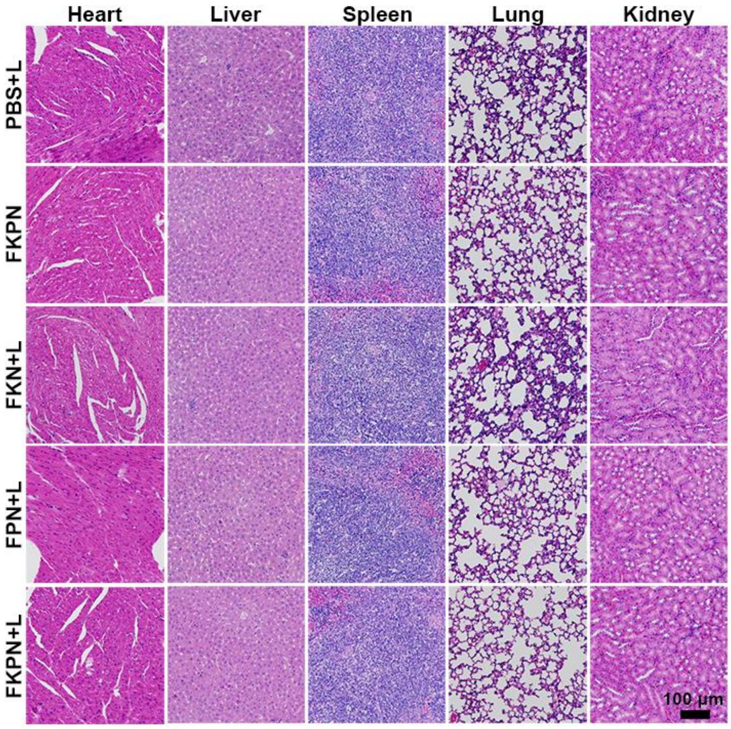

Supplementary Figure 50. Biosafety assessment after different treatments. Micrographs of H&E stained main organs (including Heart, liver, spleen, lung, kidney) of bilateral 4T1 tumor-bearing mice with different treatments. Representative of n = 3 independent experiments.

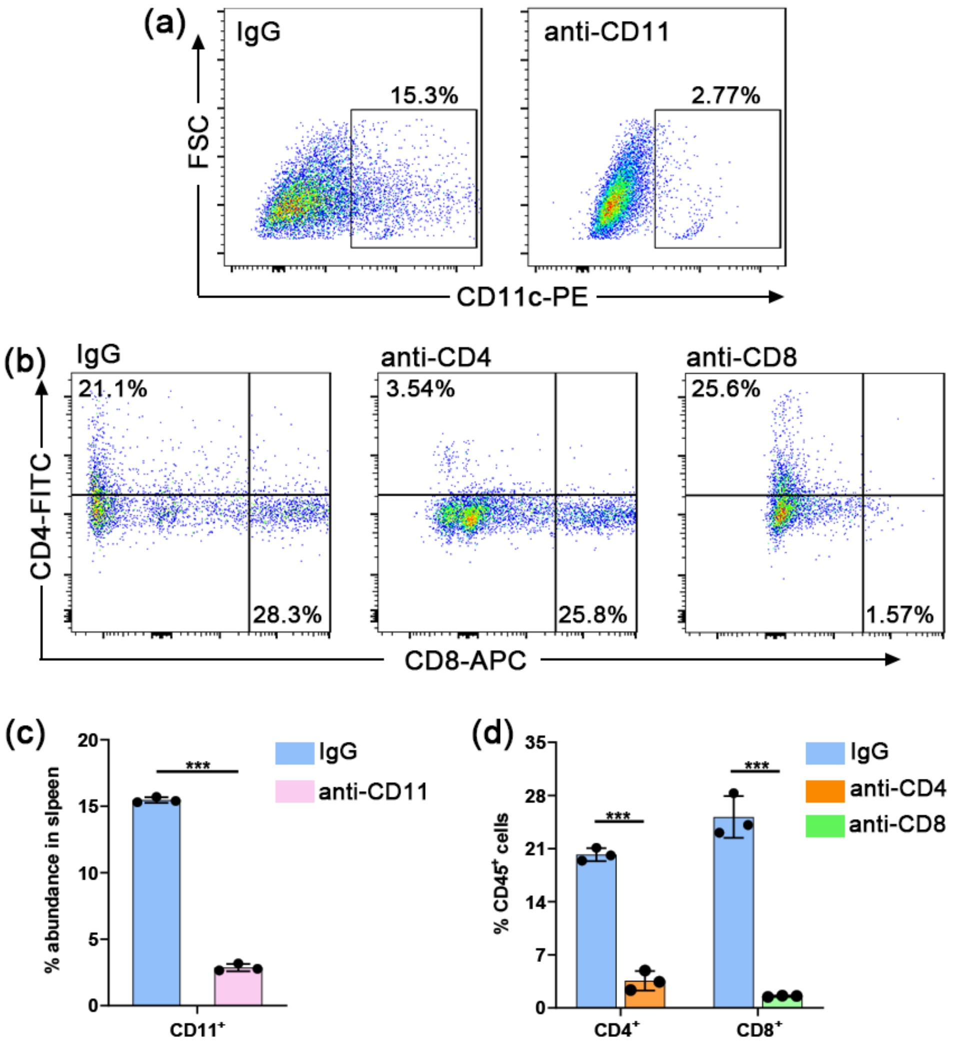

Supplementary Figure 51. Immune cell blocking. (a) Flow cytometry analysis of DC depletion with anti-CD11 antibodies. (b) Flow cytometry analysis of $CD4^+$ or $CD8^+$ depletion with anti-CD4 antibodies and anti-CD8 antibodies. Quantification of (c) DC and (d) $CD4^+$ T cell as well as $CD8^+$ T cell depletion. Data were presented as mean ± SD (n = 3 mice). Significance was assessed using Student's t test (two-tailed). P values: 3.51468E-07 for quantification of DC depletion in IgG group vs in anti-CD11 group; 4.86676E-05 for quantification of $CD4^+$ T cell depletion in IgG group vs in anti-CD4 group; 0.00012062 for quantification of $CD8^+$ depletion in IgG group vs in anti-CD8 group. (***$p < 0.001$). Source data are provided as a Source Data file.

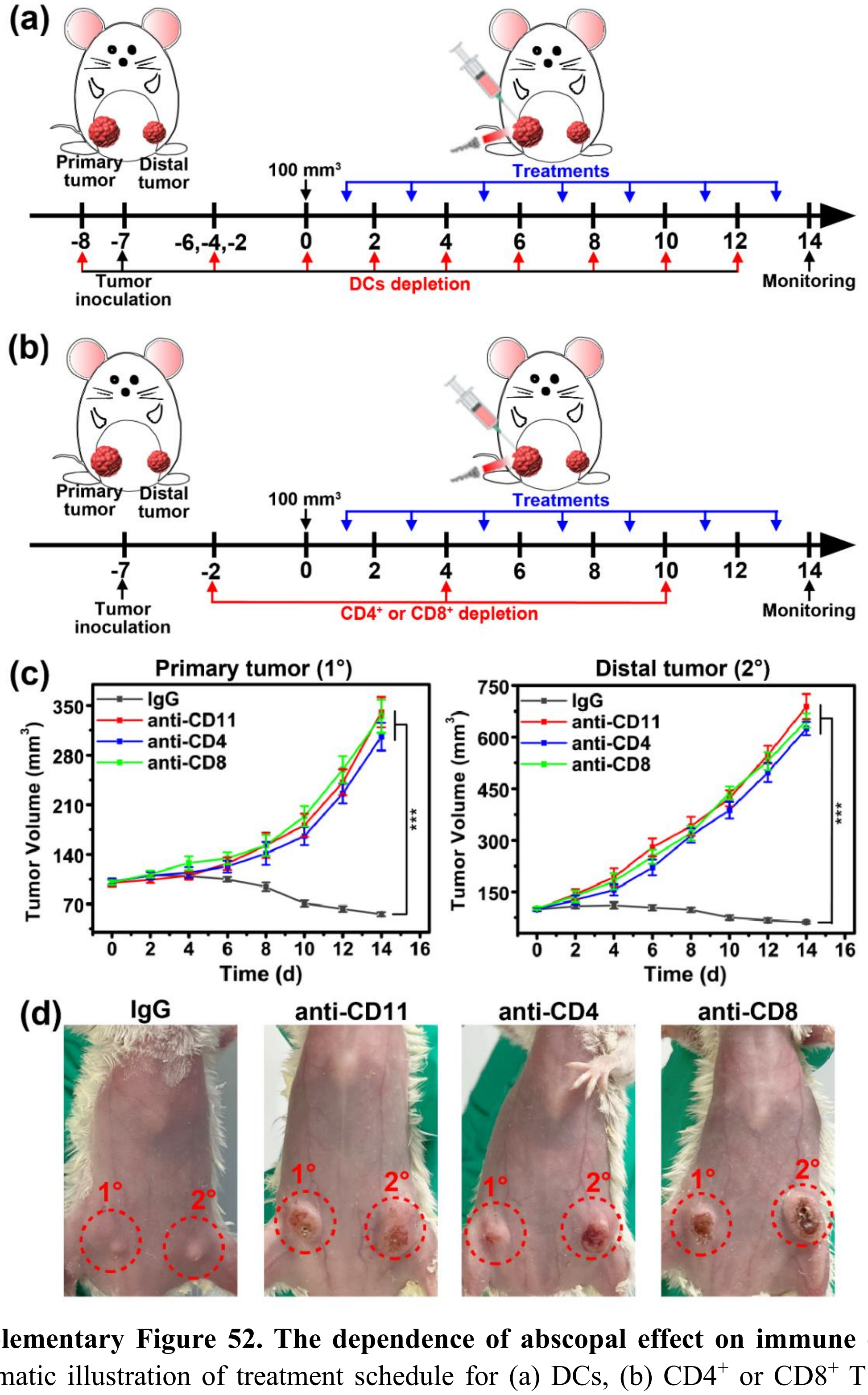

Supplementary Figure 52. The dependence of abscopal effect on immune cells. Schematic illustration of treatment schedule for (a) DCs, (b) $CD4^+$ or $CD8^+$ T cell-depleted 4T1 bilateral breast cancer BALB/c mouse model. (c) Primary and distant tumors growth curves of mice in different groups after treatments. Data were presented as mean ± SD (n = 6 mice). Significance was assessed using Student's t test (two-tailed). P values: 1.2324E-17 for primary tumor volume of mice in IgG group vs in anti-CD11, anti-CD4 and anti-CD8 groups; 2.65615E-15 for distant tumor volume of mice in IgG group vs in anti-CD11, anti-CD4 and anti-CD8 groups. (***p < 0.001). (d) The representative photographs on day 14 of bilateral 4T1 tumor-bearing mice after treatments. Source data are provided as a Source Data file.

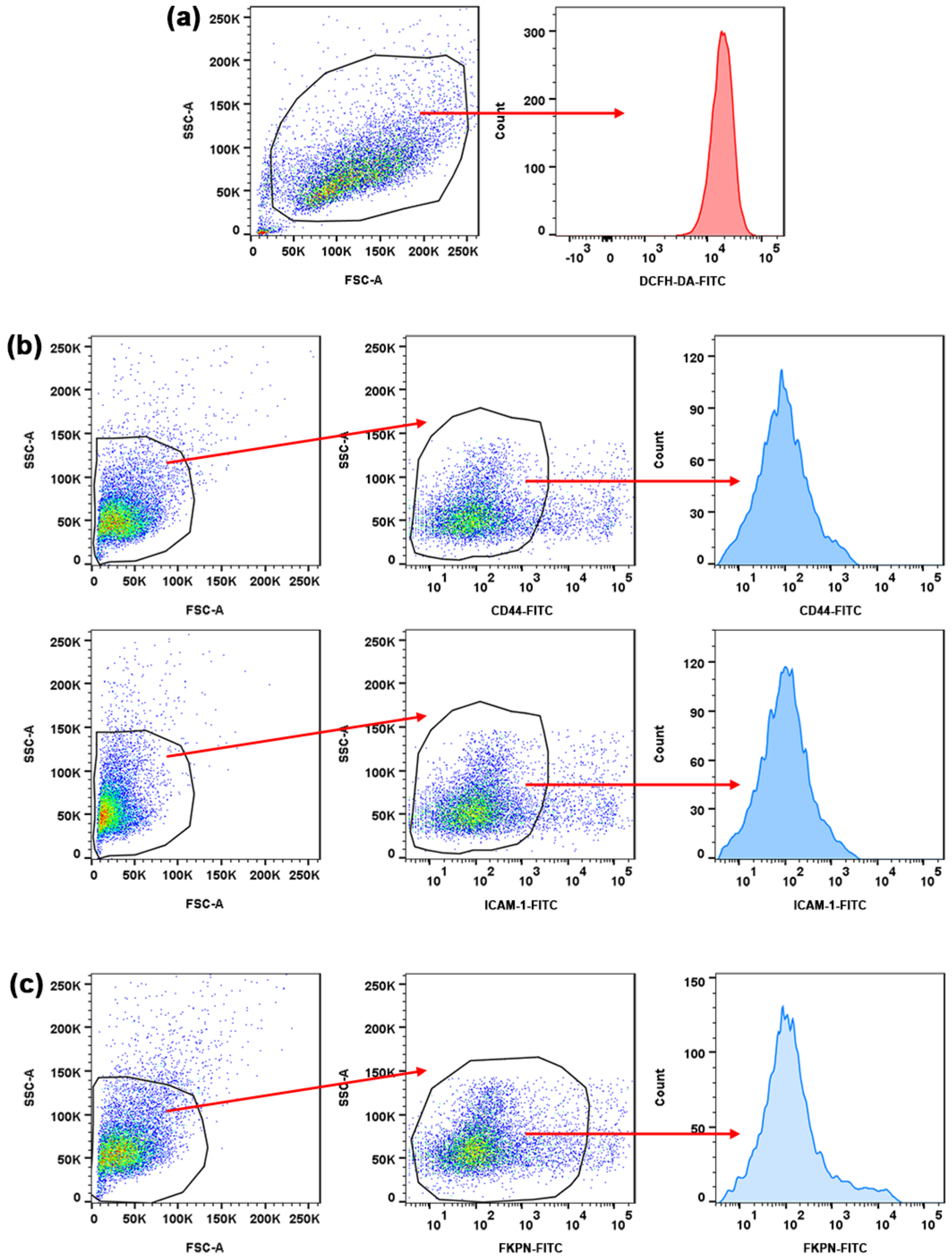

Supplementary Figure 53. Flow cytometry gating strategies. (a) The Gating strategy for experiments in Figure 3d. (b) The Gating strategies for experiments in Supplementary Figures 23a and c. (c) The Gating strategies for experiments in Supplementary Figure 25.

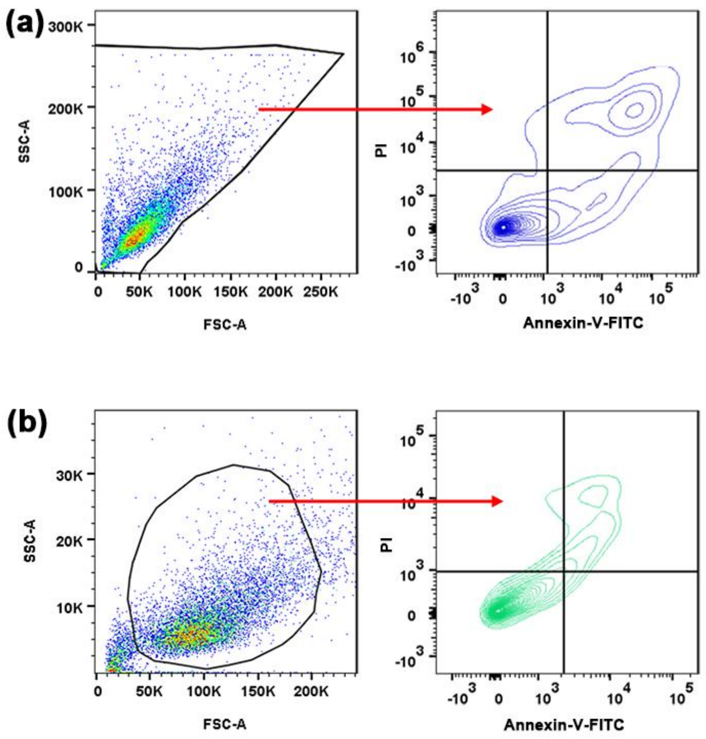

Supplementary Figure 54. Flow cytometry gating strategies. (a) The Gating strategy for experiments in Figure 4c. (b) The Gating strategies for experiments in Supplementary Figure 37b.

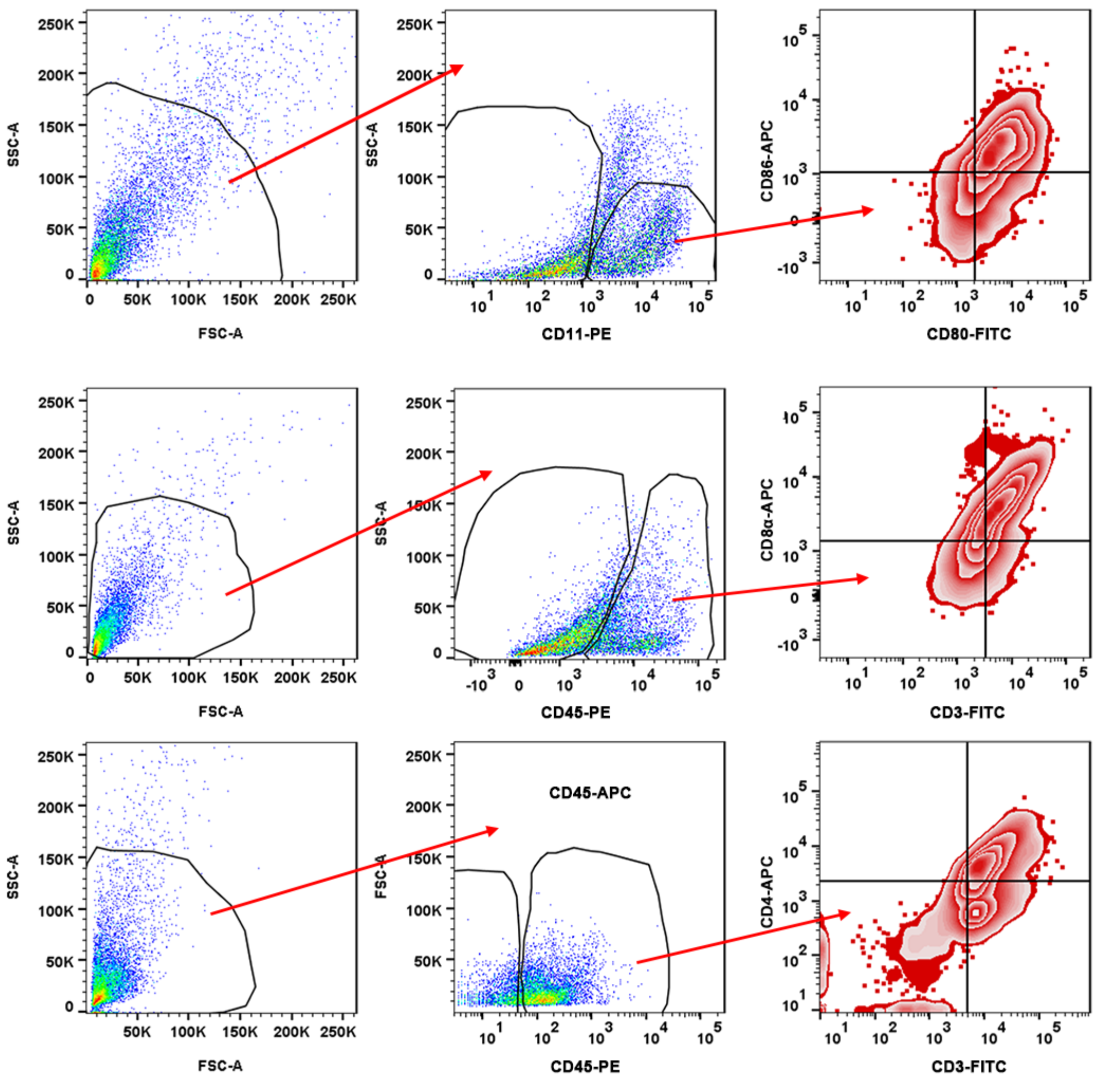

Supplementary Figure 55. Flow cytometry gating strategies for experiments in Figures 4d-f.

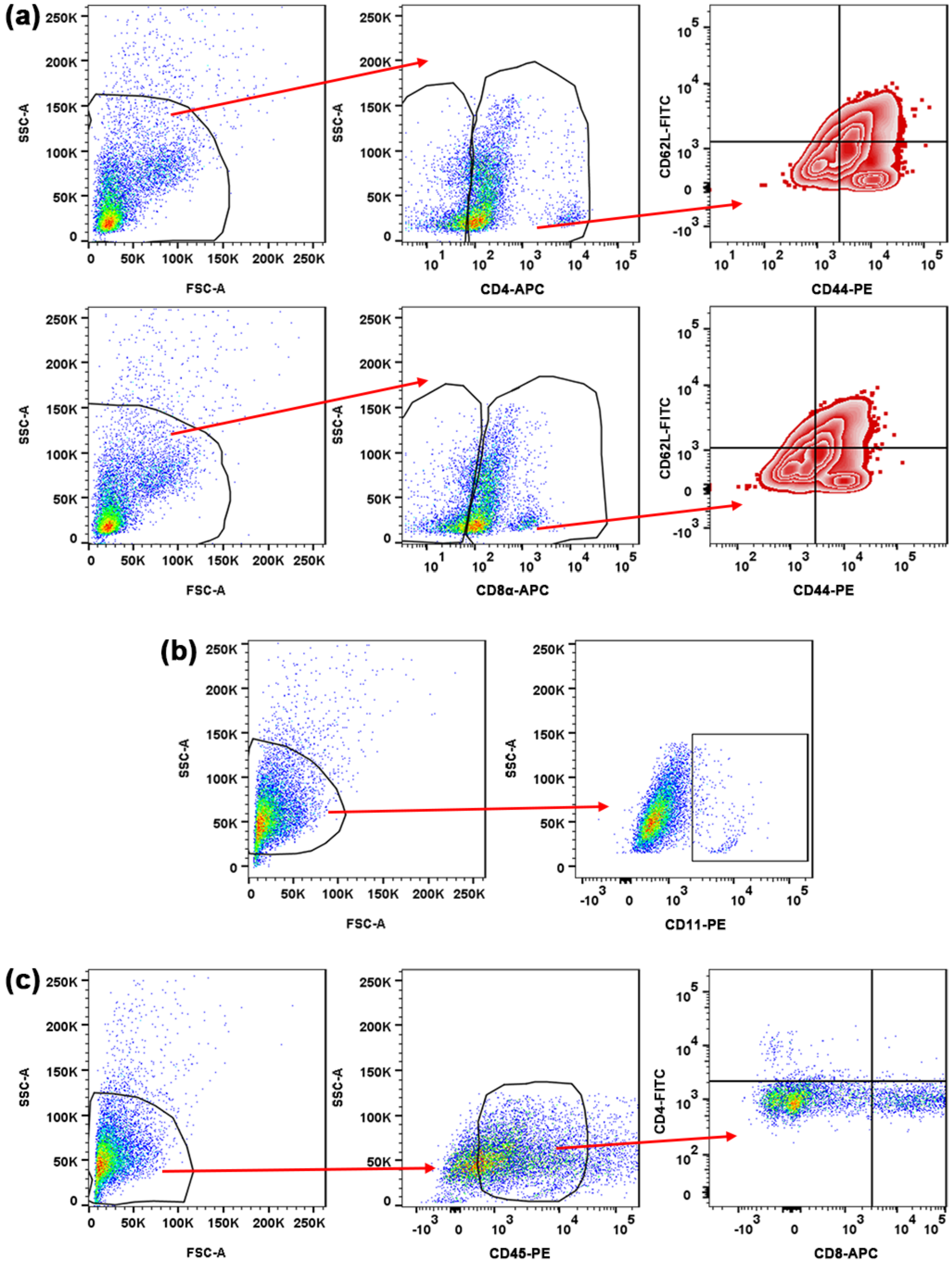

Supplementary Figure 56. Flow cytometry gating strategies. (a) The Gating strategy for experiments in Supplementary Figure 45a. (b) The Gating strategies for experiments in Supplementary Figure 51a. (c) The Gating strategies for experiments in Supplementary Figure 51b.
